# Posthospitalization COVID-19 cognitive deficits at 1 year are global and associated with elevated brain injury markers and gray matter volume reduction

**DOI:** 10.1038/s41591-024-03309-8

**Published:** 2024-09-23

**Authors:** Greta K. Wood, Brendan F. Sargent, Zain-Ul-Abideen Ahmad, Kukatharmini Tharmaratnam, Cordelia Dunai, Franklyn N. Egbe, Naomi H. Martin, Bethany Facer, Sophie L. Pendered, Henry C. Rogers, Christopher Hübel, Daniel J. van Wamelen, Richard A. I. Bethlehem, Valentina Giunchiglia, Peter J. Hellyer, William Trender, Gursharan Kalsi, Edward Needham, Ava Easton, Thomas A. Jackson, Colm Cunningham, Rachel Upthegrove, Thomas A. Pollak, Matthew Hotopf, Tom Solomon, Sarah L. Pett, Pamela J. Shaw, Nicholas Wood, Neil A. Harrison, Karla L. Miller, Peter Jezzard, Guy Williams, Eugene P. Duff, Steven Williams, Fernando Zelaya, Stephen M. Smith, Simon Keller, Matthew Broome, Nathalie Kingston, Masud Husain, Angela Vincent, John Bradley, Patrick Chinnery, David K. Menon, John P. Aggleton, Timothy R. Nicholson, John-Paul Taylor, Anthony S. David, Alan Carson, Ed Bullmore, Gerome Breen, Adam Hampshire, COVID-CNS Consortium, Greta K. Wood, Kukatharmini Tharmaratnam, Cordelia Dunai, Franklyn N. Egbe, Bethany Facer, Henry C. Rogers, Peter J. Hellyer, Gursharan Kalsi, Edward Needham, Ava Easton, Thomas A. Jackson, Colm Cunningham, Rachel Upthegrove, Matthew Hotopf, Tom Solomon, Sarah L. Pett, Pamela J. Shaw, Nicholas Wood, Neil A. Harrison, Peter Jezzard, Steven Williams, Fernando Zelaya, Simon Keller, Nathalie Kingston, Masud Husain, Angela Vincent, Patrick Chinnery, David K. Menon, John P. Aggleton, John-Paul Taylor, Anthony S. David, Alan Carson, Gerome Breen, Adam Hampshire, Ali M. Alam, Ammar Al-Chalabi, Christopher M. Allen, Jay Amin, Cherie Armour, Mark R. Baker, Suzanne Barrett, Neil Basu, Rahul Batra, Laura Benjamin, Alex Berry, Richard A. I. Bethlehem, Bethan Blackledge, Sarah A. Boardman, John Bradley, David P. Breen, Judith Breuer, Matthew Broome, Ed Bullmore, Matthew Butler, Hannah Castell, Jonathan Cavanagh, David Christmas, David M. Christmas, Jonathan R. I. Coleman, Alaistair Coles, Ceryce Collie, Nadine Cossette, David Cousins, Alastair Darby, Nicholas Davies, Sylviane Defres, Katherine C. Dodd, Alex Dregan, Eugene Duff, Mark A. Ellul, Nikos Evangelou, Peter M. Fernandes, Richard Francis, Ian Galea, Afagh Garjani, Lily George, Valentina Giunchiglia, Kiran Glen, Rebecca Gregory, Michael Griffiths, Victoria Grimbly, Alexander Grundmann, Savini Gunatilake, Shahd H. M. Hamid, Marc Hardwick, Jade D. Harris, Ewan Harrison, Paul J. Harrison, Monika Hartmann, Claire Hetherington, Orla Hilton, Julian Hiscox, Eva Maria Hodel, Angela E. Holland, Yun Huang, Stella Hughes, Sarosh Irani, Thomas M. Jenkins, Johan Kallberg Zvrskovec, Sandar Kyaw, Gabriella Lewis, James B. Lilleker, Michael P. Lunn, Claire L. MacIver, Daniel Madarshahian, Parisa Mansoori, Naomi Martin, Gavin McDonnell, Emily McGlinchey, Stephen McKeever, Ryan McIlwaine, Andrew M. McIntosh, Karla L. Miller, Dina Monssen, Christopher M. Morris, Ciaran Mulholland, Akshay Nair, Virginia Newcombe, Nathalie Nicholas, Timothy R. Nicholson, Ronan O’Malley, Obioma Orazulume, Marlies Ostermann, Alish Palmos, Arvind Patel, Sharon Peacock, Sophie L. Pendered, Thomas A. Pollak, Angela Roberts, Silvia Rota, Rustam Al-Shahi Salman, Merna Samuel, Brendan F. Sargent, Stephen J. Sawcer, Adam W. Seed, Scott Semple, Rajish S. K. Shil, Adam Sieradzki, Bhagteshwar Singh, Craig J. Smith, Jacqueline Smith, Stephen M. Smith, Leonie Taams, Arina Tamborska, Rhys H. Thomas, Emma Thomson, William Trender, Zain-Ul-Abideen Ahmad, Jonathan Underwood, Tonny Veenith, Annalena Venneri, Daniel J. van Wamelen, Guy Williams, Sui Hsien Wong, Michael S. Zandi, Benedict D. Michael, Stella-Maria Paddick, E. Charles Leek, Benedict D. Michael, Stella-Maria Paddick, E. Charles Leek

**Affiliations:** 1 Department of Clinical Infection, Microbiology & Immunology, Institute of Infection, Veterinary and Ecological Sciences, University of Liverpool, Liverpool, UK; 2 Liverpool University Hospitals NHS Foundation Trust, Liverpool, UK; 3 Department of Psychiatry, Warneford Hospital, University of Oxford, Oxford, UK; 4 Wellcome Centre for Integrative Neuroimaging, FMRIB, Nuffield Department of Clinical Neurosciences, Oxford University, Oxford, UK; 5 Social, Genetic and Developmental Psychiatry Centre, Institute of Psychiatry, Psychology & Neuroscience, King’s College London, London, UK; 6 Department of Health Data Science, Institute of Population Health, University of Liverpool, Liverpool, UK; 7 NIHR Health Protection Research Unit (HPRU) in Emerging and Zoonotic Infections, Institute of Infection, Veterinary and Ecological Sciences, University of Liverpool, Liverpool, UK; 8 Department of Pharmacology and Therapeutics, Institute of Systems, Molecular and Integrative Biology, University of Liverpool, Liverpool, UK; 9 National Centre for Register-based Research, Aarhus Business and Social Sciences, Aarhus University, Aarhus, Denmark; 10 Department of Pediatric Neurology, Charité - Universitätsmedizin Berlin, Berlin, Germany; 11 Department of Neuroimaging, Institute of Psychiatry, Psychology & Neuroscience, King’s College London, London, UK; 12 Parkinson’s Foundation Center of Excellence, King’s College Hospital, London, UK; 13 Department of Neurology; Centre of Expertise for Parkinson & Movement Disorders, Donders Institute for Brain, Cognition and Behaviour, Radboud University Medical Centre, Nijmegen, The Netherlands; 14 Department of Psychology, University of Cambridge, Cambridge, UK; 15 Department of Brain Sciences, Imperial College London, London, UK; 16 Department of Clinical Neurosciences, University of Cambridge, Cambridge, UK; 17 Encephalitis International, Malton, UK; 18 Institute of Inflammation and Ageing, University of Birmingham, Birmingham, UK; 19 School of Biochemistry and Immunology, Trinity Biomedical Sciences Institute, Trinity College Dublin, Dublin, Ireland; 20 Institute for Mental Health, School of Psychology, University of Birmingham, Birmingham, UK; 21 Department of Psychosis Studies, Institute of Psychiatry, Psychology & Neuroscience, King’s College London, London, UK; 22 South London and Maudsley NHS Foundation Trust, London, UK; 23 Department of Psychological Medicine, Institute of Psychiatry, Psychology & Neuroscience, King’s College London, London, UK; 24 The Pandemic Institute, University of Liverpool, Liverpool, UK; 25 Department of Neurology, Walton Centre Foundation Trust, Liverpool, UK; 26 MRC Clinical Trials Unit, UCL, London, UK; 27 Institute of Clinical Trials and Methodology, UCL, London, UK; 28 Institute for Global Health, UCL, London, UK; 29 Division of Neuroscience, School of Medicine and Population Health, University of Sheffield, Sheffield, UK; 30 Sheffield Institute for Translational Neuroscience, NIHR Biomedical Research Centre, University of Sheffield, Sheffield, UK; 31 Department of Clinical and Movement Neurosciences, UCL Queen Square Institute of Neurology, UCL, London, UK; 32 UCL Genetics Institute, Division of Biosciences, UCL, London, UK; 33 Cardiff University Brain Research Imaging Centre, School of Medicine, Cardiff University, Cardiff, UK; 34 Wolfson Brain Imaging Centre, Department of Clinical Neurosciences, University of Cambridge, Cambridge, UK; 35 UK Dementia Research Institute, Department of Brain Sciences, Imperial College London, London, UK; 36 NIHR Maudsley Biomedical Research Centre for Mental Health, South London and Maudsley NHS Foundation Trust, London, UK; 37 Birmingham Women’s and Children’s NHS Foundation Trust, Birmingham, UK; 38 NIHR Bioresource, Cambridge University Hospitals NHS Foundation Trust, Cambridge, UK; 39 Department of Haematology, School of Clinical Medicine, University of Cambridge, Cambridge, UK; 40 Nuffield Department of Clinical Neuroscience, John Radcliffe Hospital, University of Oxford, Oxford, UK; 41 Department of Experimental Psychology, University of Oxford, Oxford, UK; 42 MRC Mitochondrial Biology Unit, University of Cambridge, Cambridge, UK; 43 Section of Perioperative, Acute, Critical Care and Emergency Medicine, Department of Medicine, University of Cambridge, Cambridge, UK; 44 School of Psychology, Cardiff University, Cardiff, UK; 45 Neuropsychiatry Research and Education Group, Institute of Psychiatry, Psychology & Neuroscience, King’s College London, London, UK; 46 Translational and Clinical Research Institute, Newcastle University, Newcastle, UK; 47 Old Age Psychiatry, Tyne and Wear NHS Trust, Newcastle, UK; 48 Department of Psychiatry, Institute of Mental Health, UCL, London, UK; 49 Centre for Clinical Brain Sciences, University of Edinburgh, Edinburgh, UK; 50 Department of Psychiatry, Institute of Behavioural and Clinical Neuroscience, University of Cambridge, Cambridge, UK; 51 Department of Old Age Psychiatry, Gateshead Health NHS Foundation Trust, Gateshead, UK; 52 Millenium Institute for Care Research (MICARE), Santiago, Chile; 53 Department of Psychology, Institute of Population Health, Institute of Life and Human Sciences, University of Liverpool, Liverpool, UK; 54 School of Psychology, University of Southampton, Southampton, UK; 55 King’s College London, London, UK; 56 University of Nottingham, Nottingham, UK; 57 University of Southampton, Southampton, UK; 58 Queen’s University Belfast, Belfast, UK; 59 Newcastle University, Newcastle, UK; 60 Northern Health and Social Care Trust, Antrim, UK; 61 University of Glasgow, Glasgow, UK; 62 University College London, London, UK; 63 Salford Royal, Northern Care Alliance NHS Foundation Trust, Manchester, UK; 64 University of Dundee, Dundee, UK; 65 University of Cambridge, Cambridge, UK; 66 Royal Infirmary of Edinburgh, NHS Lothian, Edinburgh, UK; 67 University of Liverpool, Liverpool, UK; 68 Imperial College London, London, UK; 69 Liverpool School of Tropical Medicine, Liverpool, UK; 70 University of Manchester, Manchester, UK; 71 The Stroke Association, London, UK; 72 Sheffield Teaching Hospitals NHS Foundation Trust, Sheffield, UK; 73 Royal Stoke University Hospital, Stoke-On-Trent, UK; 74 Nottingham University Hospital, Nottingham, UK; 75 Belfast Health and Social Care Trust, Belfast, UK; 76 Institute of Mental Health, Nottingham, UK; 77 Cardiff University, Cardiff, UK; 78 National Institute for Health Research (NIHR) Bioresource, London, UK; 79 Aintree University Hospital, Liverpool University Hospitals NHS Foundation Trust, Liverpool, UK; 80 University of Edinburgh, Edinburgh, UK; 81 COVID-CNS Consortium, Liverpool, UK; 82 University of Sheffield, Sheffield, UK

**Keywords:** Outcomes research, Encephalopathy, Predictive markers, Chronic inflammation, Viral infection

## Abstract

The spectrum, pathophysiology and recovery trajectory of persistent post-COVID-19 cognitive deficits are unknown, limiting our ability to develop prevention and treatment strategies. We report the 1-year cognitive, serum biomarker and neuroimaging findings from a prospective, national study of cognition in 351 COVID-19 patients who required hospitalization, compared with 2,927 normative matched controls. Cognitive deficits were global, associated with elevated brain injury markers and reduced anterior cingulate cortex volume 1 year after COVID-19. Severity of the initial infective insult, postacute psychiatric symptoms and a history of encephalopathy were associated with the greatest deficits. There was strong concordance between subjective and objective cognitive deficits. Longitudinal follow-up in 106 patients demonstrated a trend toward recovery. Together, these findings support the hypothesis that brain injury in moderate to severe COVID-19 may be immune-mediated, and should guide the development of therapeutic strategies.

## Abstract (web summary)

A national prospective study of patients requiring hospitalization for COVID-19 demonstrates global cognitive deficits at 1 year, associated with elevated brain injury markers and reduced gray matter volume.

## Main

Cognitive deficits have been widely reported in postacute COVID-19 patients across the respiratory disease severity spectrum; however, their recovery trajectory and pathophysiology remain unknown^1,2^. The most severely impacted patients are likely to be those with symptoms of and clinical evidence for neurological or psychiatric complications secondary to COVID-19 (ref. ^3^). However, most previous studies have not included these patients despite such complications being present in up to one-third of patients in the 6 months following COVID-19 diagnosis, including diagnoses such as stroke, movement disorders and psychosis^4^. Early data suggested that the most common acute neurological complication of COVID-19 was encephalopathy, overlapping with delirium and subacute delirium in the context of COVID-19 (refs. ^5,6^). Many of the extant studies that have used optimal or multidomain measures of cognitive performance have not also examined biological substrates^7–9^. Similarly, there are few neuroimaging studies that combine quality neuroimaging measures and the assessment of cognition across multiple cognitive domains, with the utilization of sensitive, precise and objective assessments, in both post-COVID individuals and appropriately matched controls^10–13^. In addition, there are scarce studies with follow-up cognitive and neuroimaging data to allow understanding of recovery trajectories and prognostic markers^8,9,14^.

Early evidence suggested that COVID-19 patients primarily suffered from a dysexecutive syndrome during acute infection^15^. However, the domain-specific pattern of cognitive impairment in the postacute phase, commonly defined as beyond 3 months after COVID-19 symptom onset^16,17^, has not been well characterized^18,19^. Similarly, the biological basis of these objective cognitive deficits remains unclear, particularly the degree of brain injury and associated changes in structural neuroimaging. Given that COVID-19 is very rarely neuroinvasive, with little robust evidence for SARS-Cov-2 virions in the brain^17^, the impact on the brain is hypothesized to be via immune-mediated para- and postinfectious phenomena^20,21^, or else indirect effects via neuropsychiatric, psychological and social consequences of illness and the pandemic more generally. The para-infectious brain insult demonstrated in COVID-19 is unlikely to be unique to SARS-CoV-2 infection given that similar findings have been demonstrated in other systemic infections and critical illness^22–24^ and therefore improved understanding of postacute cognitive impairment in this setting may be translatable to other clinical cohorts.

Ultimately, the current lack of evidence limits our ability to advise and manage patients with ongoing cognitive symptoms that can have a marked impact on quality of life and healthcare systems^25–27^. There is an urgent need to comprehensively study COVID-19 patients including in-depth clinical, biological and cognitive phenotyping, as well as longitudinal follow-up. The COVID-19 Clinical Neuroscience Study (COVID-CNS) is a prospective, national study of the neurological and psychiatric complications of COVID-19. This analysis aims to characterize postacute cognitive impairment and explore the role of serum and neuroimaging biomarkers in adults hospitalized with COVID-19, with and without acute clinical neurological and psychiatric complications. Analyses were conducted according to a preregistered statistical analysis plan^28^ to test the following hypotheses:

1. COVID-19 is associated with postacute objectively measurable cognitive deficits.
2. Certain cognitive domains are more greatly impaired than others. Executive function will be disproportionately impaired in relation to accuracy and reaction time.
3. Cognitive deficits correlate with age, World Health Organization (WHO) COVID-19 disease severity, presence of an acute neurological or psychiatric complication, multimorbidity and mental health comorbidities, Rockwood Clinical Frailty Scale, and acute serum inflammatory markers.
4. Educational attainment and previous treatment with dexamethasone during acute illness may be protective.
5. Postacute cognitive deficits are associated with structural volumetric changes on magnetic resonance imaging (MRI).

## Results

### Study population

**Fig. 1:**
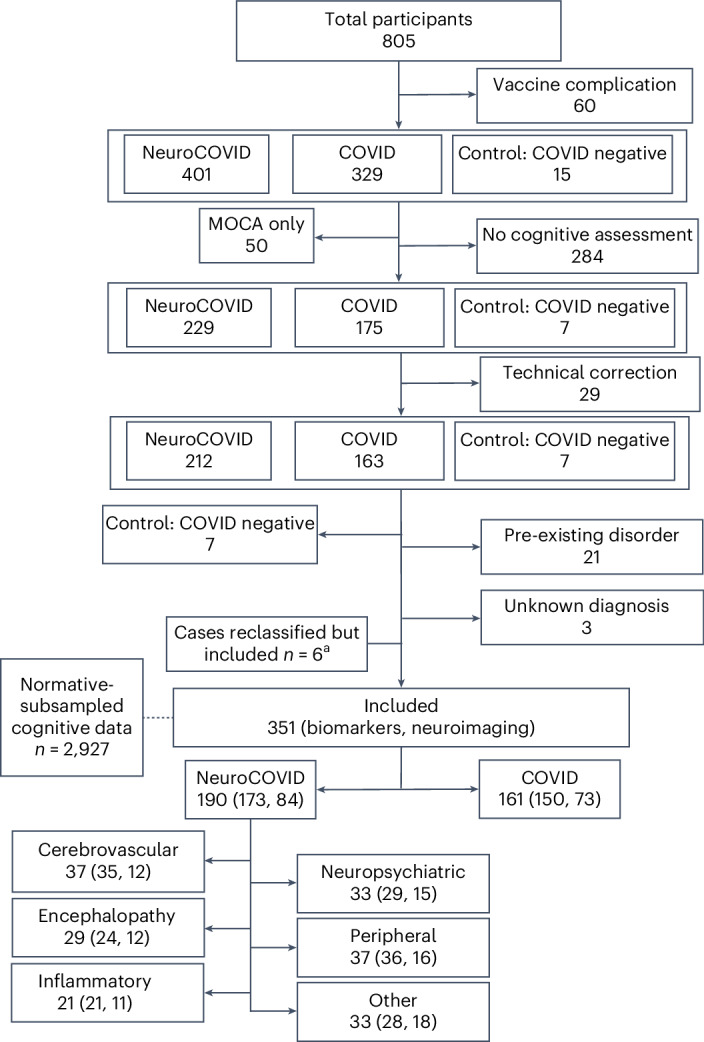
Flow diagram of patients included from the COVID-CNS. Nationally at least 16,279 patients were screened of whom at least 2,712 were eligible. Matched community data were collected separately and held in a large normative database. ‘Other’ includes autonomic dysfunction^3^, cerebral hypoxic injury^2^, headache^6^, headache and fatigue^2^, hyperkinetic movement disorder^2^, Parkinsonian movement disorder^2^, seizures^7^ and speech and sensory^1^. ^a^Six patients with ‘anosmia/ageusia’ reclassified as COVID from NeuroCOVID. The parentheses show *n* with biomarkers, *n* with neuroimaging. MOCA, Montreal Cognitive Assessment.

The analysis included 351 COVID-CNS participants and a normative comparator group of 2,927 subsampled age, sex, first language and education level matched community controls (Fig. 1). Participants were identified if they did not have a previous neurological diagnosis, and were assessed at a single postacute appointment median (interquartile range (IQR)) 384 (155–574) days after COVID-19, including cognitive testing, self-reported measures, neuroimaging and serum sampling. Within the COVID-CNS cohort, the median (IQR) age was 54 (44–63) years, 202 (58%) were male, 271 of 348 (78%) were of white ethnicity and 89 of 311 (29%) had severe SARS-CoV-2 disease symptoms, as per the WHO clinical severity scale (Table 1)^29^. In total, 57 of 294 (19%) patients had been vaccinated with two doses against SARS-CoV-2 at least two weeks before COVID-19; and 257 of 306 (84%) of patients had received two doses by the time of their postacute assessment. Some 190 of 351 (54%) patients had a neurological or psychiatric complication associated with their COVID-19 illness (the NeuroCOVID group with six clinical diagnostic subgroups) and 161 of 351 (46%) had no neurological complication (the COVID group) (Fig. 1). Compared with the COVID group, the NeuroCOVID group were more likely to have mild COVID-19, were assessed earlier post-COVID-19 and had higher self-rated scores for mental health measures (Table 1).

**Table 1:** Demographics of cohort, comparing NeuroCOVID and COVID groups

Characteristic	Overall (*N* = 351)^a^	NeuroCOVID (*N* = 190)^a^	COVID (*N* = 161)^*a*^	*P* value^b^
Age (years)	54 (44, 63)	54 (43, 63)	54 (44, 62)	0.973
Sex				0.061
Female	149 (42)	72 (38)	77 (48)	
Male	202 (58)	118 (62)	84 (52)	
First language				0.4
English	307 (87)	169 (89)	138 (86)	
Other	44 (13)	21 (11)	23 (14)	
Level of education				0.022
None of the below	27 (7.7)	10 (5.3)	17 (11)	
College or university degree	166 (47)	83 (44)	83 (52)	
A levels/AS levels or equivalent (school/vocational)	30 (8.5)	17 (8.9)	13 (8.1)	
O levels/GCSEs or equivalent (school/vocational)	78 (22)	47 (25)	31 (19)	
CSEs or equivalent (school/vocational)	20 (5.7)	13 (6.8)	7 (4.3)	
NVQ or HND or HNC or equivalent (school/vocational)	23 (6.6)	15 (7.9)	8 (5.0)	
Other professional qualifications (school/vocational)	7 (2.0)	5 (2.6)	2 (1.2)	
Premorbid Clinical Frailty Scale				0.013
Managing well (1–3)	261 (91)	137 (87)	124 (96)	
Mild (4–5)	23 (8.0)	18 (11)	5 (3.9)	
Moderate–severe (6–8)	3 (1.0)	3 (1.9)	0 (0)	
Unknown	64	32	32	
WHO COVID-19 Severity				<0.001
Ambulatory mild disease	84 (27)	61 (39)	23 (15)	
Hospitalized: moderate	138 (44)	47 (30)	91 (59)	
Hospitalized: severe	89 (29)	49 (31)	40 (26)	
Unknown	40	33	7	
Days since COVID-19	384 (155, 574)	341 (179, 463)	473 (138, 728)	0.005
Unknown	41	32	9	
Admission date				0.026
1 March 2020 to 31 August 2020	93 (29)	49 (28)	44 (30)	
1 September 2020 to 28 February 2021	107 (33)	64 (37)	43 (29)	
1 March 2021 to 31 August 2021	42 (13)	28 (16)	14 (9.5)	
1 September 2021 to 28 February 2022	61 (19)	23 (13)	38 (26)	
1 March 2022 to 31 August 2022	17 (5.3)	8 (4.7)	9 (6.1)	
Unknown	31	18	13	
Previous COVID-19 vaccination^c^	57 (19)	31 (20)	26 (18)	0.7
Unknown	57	37	20	
Acute steroid treatment	148 (48)	72 (45)	76 (52)	0.2
Unknown	45	29	16	
Memory concerns	164 (47)	98 (52)	66 (41)	0.048
Unknown	2	1	1	
PHQ-9 score	5.0 (2.0, 10.0)	6.0 (2.0, 10.0)	4.0 (1.0, 9.5)	0.042
Unknown	36	18	18	
GAD-7 score	3.0 (0, 8.0)	3.0 (0.5, 8.0)	2.5 (0, 6.0)	0.13
Unknown	30	15	15	
PCL-5 score	10 (2, 22)	12 (4, 24)	6 (1, 19)	0.002
Unknown	99	49	50	
Cognitron Global Score	−0.92 (−1.83, −0.26)	−1.11 (−2.00, −0.35)	−0.83 (−1.70, −0.19)	0.063
Cognitron Accuracy	−0.89 (−1.58, −0.21)	−1.04 (−1.67, −0.29)	−0.75 (−1.53, −0.09)	0.050
Cognitron RT	0.61 (−0.05, 1.54)	0.70 (−0.04, 1.78)	0.50 (−0.06, 1.39)	0.11

^a^ Values are shown as median (IQR) or *n* (%).

^b^ Two-sided Wilcoxon rank sum test, Pearson’s chi-squared test or Fisher’s exact test.

^c^ Binary. ‘Yes’ if the patient had received at least two doses of SARS-CoV-2 vaccine with the most recent dose at least two weeks before COVID-19; ‘No’ otherwise.

A level, Advanced level; AS level, Advanced Subsidiary level; CSE, Certificate of Secondary Education; GCSE, General Certificate of Secondary Education; HNC, Higher National Certificate; HND, Higher National Diploma; NVQ, National Vocational Qualifications; O level, Ordinary level.

### Cognition

#### Hypothesis 1: cognitive deficits

**Fig. 2:**
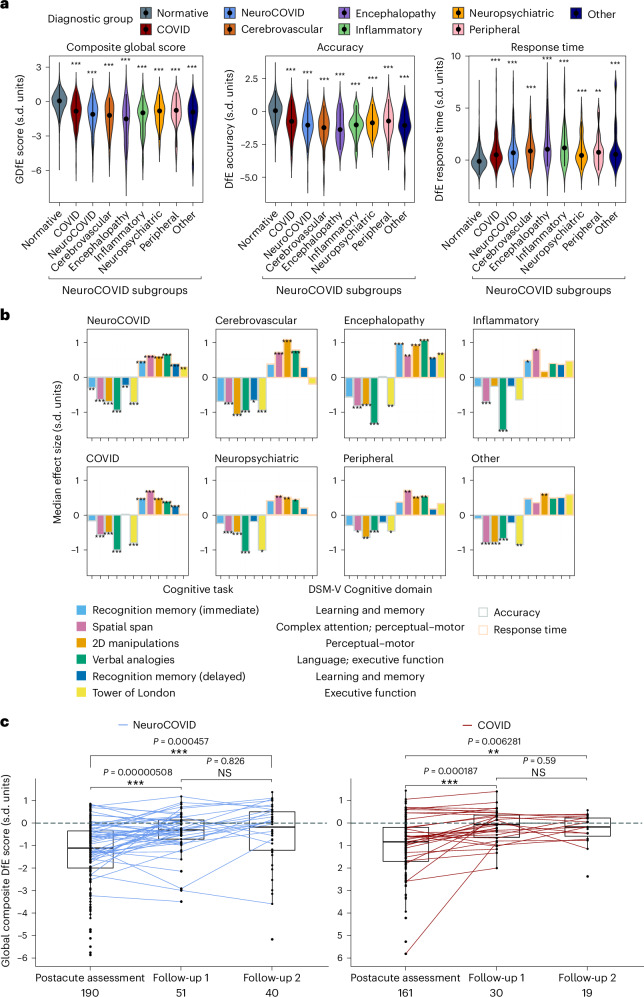
Cognitive scores and recovery trajectories. **a**, Violin plot of DfE cognition scores by diagnostic group including median (IQR) (black). Statistics compare each group with normative data, *n* = normative (2,927), cerebrovascular^37^, encephalopathy^29^, inflammatory^21^, neuropsychiatric^33^, peripheral^37^ and other^33^. Exact *P* values are listed in Supplementary Table 8. **b**, Pattern of deficits in clinical groups by median DfE accuracy and responsive time minus matched community controls across six cognitive tasks. Exact effect sizes and *P* values in listed Supplementary Table 9. **c**, Recovery trajectories in NeuroCOVID and COVID patients following postacute assessment. A black dot indicates a single observation, lines connect paired observations between postacute assessment and follow-up 1, and follow-up 1 and follow-up 2. Center line, median; box limits, upper and lower quartiles; whiskers, 1.5× IQR; the dashed line shows normal cognition; the numbers under the *x* axis show *n* for each assessment. *P < 0.05, **P < 0.01, ***P < 0.001 two-sided Mann–Whitney *U*-test, adjusted for multiple comparisons based on the FDR approach in **a** and **b** (adjusted for *n* = 8 and *n* = 12 comparisons, respectively). 2D, two-dimensional; NS, nonsignificant.

Patients in all groups were significantly less accurate and slower in their responses than would be expected based upon their demographics compared with subsampled normative data (Fig. 2a). The lowest Global Deviation from Expected (GDfE (IQR)) scores were seen in patients who had had encephalopathy (−1.51 (2.87)) and to a lesser extent those who had had cerebrovascular (−1.20 (1.75)) or inflammatory (−0.98 (1.55)) complications (Fig. 2a). Before COVID-19 illness, 11 of 137 (8%) NeuroCOVID and 15 of 152 (10%) COVID patients were concerned about their memory, increasing to 84 of 139 (60%) and 66 of 150 (44%) after COVID-19 illness respectively, of whom 35 of 82 (43%) and 45 of 66 (68%), respectively, perceived their memory problems to be progressive. Memory concerns were associated with greater objective deficits in median (IQR) GDfE scores in both NeuroCOVID (−1.26 (1.51) versus −0.76 (1.83), Mann–Whitney *U* = 5,444, estimate (confidence interval (CI)) 0.488 (0.119–0.841), effect size = 0.19, *P* = 0.009) and COVID groups (−1.30 (1.78) versus −0.59 (1.39), *U* = 4,175, estimate (CI) 0.691 (0.334–1.06), effect size = 0.29, *P* < 0.001). The positive predictive value of memory concerns for a GDfE score below expected (<0) and for poor cognitive performance (GDfE < −1) were similar in the NeuroCOVID (0.92 and 0.58), and COVID (0.89 and 0.59) groups respectively.

#### Hypothesis 2: cognitive domains

Analysis of individual tasks identified global impairment across all cognitive domains in both accuracy and response time (RT) for all clinical diagnostic groups (Fig. 2b and Extended Data Table 1)—and no evidence for domain-specific deficits. In addition, this pattern of generalized cognitive impairment did not vary significantly according to the clinical diagnostic group (effect size, eta^2^ = 0.04, *P* = 0.151).

#### Recovery

In total, 106 patients completed at least one follow-up assessment. Follow-up 1 was completed by 51 NeuroCOVID and 30 COVID patients at a median (IQR) of 111 (102–163) days after their postacute appointment. Of these participants, 48 of 51 NeuroCOVID and 27 of 30 COVID patients had serum sampling for brain injury markers at the original postacute appointment, and 21 of 51 and 15 of 30 respectively, had neuroimaging. The NeuroCOVID and COVID groups at follow-up 1 were of similar median (IQR) age (57 (46–65) and 53 (48–60) years) and sex (31 of 51 (61%) and 20 of 30 (67%) male respectively) as the cohort as a whole, but both groups had higher median (IQR) GDfE (−0.61 (−1.34 to −0.16) and −0.60 (1.08 to 0.075)) at their initial postacute assessment (Supplementary Tables 3 and 4). In both the NeuroCOVID and COVID groups, there was evidence of recovery in cognitive performance comparing the postacute assessment to both follow-up 1 and follow-up 2, but not between follow-up 1 and follow-up 2 (Fig. 2c). Multiple linear regression models accounting for age and timing of COVID-19 found no significant associations with recovery in the NeuroCOVID group (coefficient of determination, *R*^2^ = 0.30, *P* = 0.66) and the COVID group (*R*^2^ = 0.23, *P* = 0.78) (Extended Data Table 2).

#### Hypotheses 3 and 4: associated clinical factors

The clinical factors associated with cognitive impairment differed in the NeuroCOVID and COVID groups (Table 2 and Extended Data Table 3). In both NeuroCOVID and COVID groups respectively, correlation matrices revealed high correlation between scores in the Patient Health Questionnaire-9 (PHQ-9) and PTSD Checklist for DSM-5 (PCL-5) (0.78, 0.79), Generalized Anxiety Disorder Assessment (GAD-7) (0.71, 0.83), Chalder Fatigue Scale physical (0.54,0.49) and mental (0.43, 0.51) subscales and subjective cognitive impairment (0.42, 0.64). There was a significant difference between the NeuroCOVID and COVID groups in terms of days between COVID-19 illness and postacute assessment (Table 1) (*U* = 9,787, estimate (CI) 96 (27–175) days, effect size = 0.16, *P* = 0.005); however, days since COVID-19 was not significantly correlated with GDfE in the NeuroCOVID group (coefficient (s.e.) = 0.00092 (0.00059)) or the COVID group −0.00018 (0.00032)).

**Table 2:** Univariate associations, clinical linear regression model and multifaceted linear regression models for GDfE score in NeuroCOVID and COVID groups

Variable	NeuroCOVID					COVID				
	Univariate^a^	Coefficient *P* value	Univariate effect size^b^	Clinical model, *n* = 93 of 190, *R*^2^ = 0.28, *P* = 0.44	Multifaceted model, *n* = 54 of 190, *R*^2^ = 0.68, *P* = 0.00064	Univariate^a^	Coefficient *P* value	Effect size	Clinical model, *n* = 89 of 161, *R*^2^ = 0.42, *P* = 0.003	Multifaceted model, *n* = 53 of 161 *R*^2^ = 0.68, *P* = 0.00051
	Coefficient (s.e.)			Multivariate estimate (s.e.)	Multivariate estimate (s.e.)	Coefficient (s.e.)			Multivariate estimate (s.e.)	Multivariate estimate (s.e.)
Clinical										
Age (years)	−0.0038 (0.0072)	0.600	−0.02	−0.010 (0.013)	−0.044 (0.017)*, *P* = 0.0113	−0.012 (0.0074)	0.117	−0.12	−0.00039 (0.013)	0.0094 (0.015)
Level of education										
Degree	0.76 (0.47)	0.104	0.07	0.35 (0.67)		0.75 (0.31)*	0.017	0.24	−0.17 (0.49)	
School, vocational	0.36 (0.46)	0.435	0.02	−0.065 (0.66)	NI	0.11 (0.32)	0.727	0.02	−0.75 (0.46)	NI
None of above (reference)										
Clinical Frailty Scale										
Mild (4–5)	−0.24 (0.34)	0.480	0.02	−0.48 (0.62)		−0.18 (0.56)	0.747	0.03	−0.073 (0.59)	
Moderate–severe (6–8)	1.04 (0.79)	0.187	0.15	NI	NI	ND		ND		NI
Admission date										
1 March 2020 to 1 September 2020	0.35 (0.35)	0.310	0.15	0.017 (1.25)	2.69 (1.71)	−0.25 (0.26)	0.350	0.09	−0.25 (1.88)	−0.93 (1.74)
1 September 2020 to 1 March 2021	−0.54 (0.33)	0.106	0.17	−1.92 (1.07)	2.60 (1.51)	−0.80 (0.26)**	0.00290	0.28	−0.39 (0.77)	−0.0011 (1.39)
1 March 2021 to 1 September 2021	−0.20 (0.38)	0.604	0.04	−1.05 (1.29)	−0.91 (1.52)	−0.47 (0.37)	0.210	0.15	1.09 (0.79)	5.60 (3.45)
1 September 2021 to 1 March 2022 (reference)										
1 September 2022	−0.37 (0.56)	0.511	0.14	−2.17 (1.61)	ND	−0.18 (0.44)	0.689	0.04	−4.71 (2.52)	1.98 (1.50)
Days since COVID-19	0.00092 (0.00059)	0.12	0.14	−0.0014 (0.0017)	0.000099 (0.0020)	−0.00018 (0.00032)	0.574	−0.0099	−0.0000079 (0.0023)	0.0018 (0.0020)
WHO COVID-19 severity										
Moderate	−0.16 (0.28)	0.566	0.06	-0.28 (0.43)	NI	0.81 (0.28)**	0.00481	0.28	−0.15 (0.43)	−0.98 (0.54)
Severe	−0.26 (0.27)	0.338	0.04	−0.51 (0.50)		0.85 (0.32)**	0.00807	0.34	0.15 (0.49)	−0.85 (0.66)
Mild (reference)										
Diagnostic group										
Cerebrovascular	−0.39 (0.32)	0.229	0.16	−0.44 (0.55)	−0.93 (0.73)	NI	NI	NI	NI	NI
Encephalopathy	−0.84 (0.35)*	0.0164	0.25	−0.78 (0.50)	0.062 (0.51)					
Inflammatory	−0.48 (0.38)	0.211	0.17	−0.24 (0.56)	−0.29 (0.57)					
Neuropsychiatric	−0.057 (0.33)	0.865	0.07	0.10 (0.54)	−0.26 (0.48)					
Other	−0.31 (0.33)	0.352	0.11	0.018 (0.50)	−0.66 (0.48)					
Peripheral (reference)										
PHQ-9 score (per unit)	−0.054 (0.017) **	0.00205	−0.20	−0.013 (0.027)	0.030 (0.030)	−0.065 (0.017)***	0.000192	−0.29	−0.063 (0.024)*, *P* = 0.0126	−0.056 (0.028)*, *P* = 0.0496
Multimorbidity	−0.051 (0.073)	0.492	−0.00094	0.086 (0.12)	0.31 (0.13)*, *P* = 0.0254	−0.16 (0.058)**	0.00734	−0.11	−0.18 (0.088)*, *P* = 0.0462	−0.20 (0.083)*, *P* = 0.0206
Steroid treatment	−0.25 (0.22)	0.257	0.08	0.59 (0.40)	NI	0.028 (0.20)	0.89	0.04	−0.15 (0.28)	NI
Brain injury markers (pg ml^−1^)										
NfL	0.00063 (0.0012)	0.61	−0.05	NI	0.033 (0.025)	−0.0014 (0.0035)	0.692	0.056	NI	0.038 (0.031)
GFAP	0.00048 (0.00088)	0.584	−0.0022	NI	0.0041 (0.0042)	−0.00041 (0.0015)	0.788	−0.030	NI	−0.0081 (0.0032)*, *P* = 0.0168
Neuroimaging										
Anterior cingulate cortex volume	0.23 (0.078)**	0.00457	0.31	NI	0.23 (0.091)*, *P* = 0.0165	0.32 (0.10)**	0.00253	0.35	NI	0.15 (0.13)

Variables included in multivariate analysis are described. NI, not included; ND, no data. Date of admission, days since COVID-19 and geographically clustered recruitment site were included in models with the interaction term Admission date: Days since COVID-19. Two-sided linear regression coefficient *P < 0.05, **P < 0.01, ***P < 0.001.

^a^GDfE represents how an individual performs compared with what would be expected based upon their age, sex, first language and level of education.

^b^Mann–Whitney *U*-test for categorical variables, Spearman’s rho for continuous variables, Pearson’s *r* for image-derived phenotypes; >0.1 small effect size, >0.3 medium effect size.

Multiple linear regression models were developed based upon complete case analysis (Table 2 and Supplementary Table 6). For the NeuroCOVID clinical model, 92% of individual data points were present and the rates of missingness in the included variables were: days since COVID-19 (17%), WHO COVID-19 severity (17%), Clinical Frailty Scale (17%), steroid treatment (15%), PHQ-9 score (9%), admission date (9%), age (0%), recruitment site (0%), diagnostic group (0%), education category (0%) and multimorbidity (0%). The NeuroCOVID clinical model (*n* = 93 of 190) did not explain a significant proportion of the variance (*R*^2^ = 0.28, *P* = 0.44).

In the COVID clinical model, 94% of individual data points were available and the missingness of included variables was: Clinical Frailty Scale (20%), PHQ-9 score (11%), steroid treatment (10%), admission date (8%), days since COVID-19 (6%), WHO COVID-19 severity (4%), age (0%), recruitment site (0%), education category (0%) and multimorbidity (0%). The COVID clinical model (*n* = 89 of 161, *R*^2^ = 0.42, *P* = 0.003) demonstrated that GDfE score was associated with symptoms of depression (effect size (s.e.) = −0.063 (0.024), *P* = 0.013) and multimorbidity (−0.18 (0.088), *P* = 0.046).

#### Serum markers: brain injury markers

**Fig. 3:**
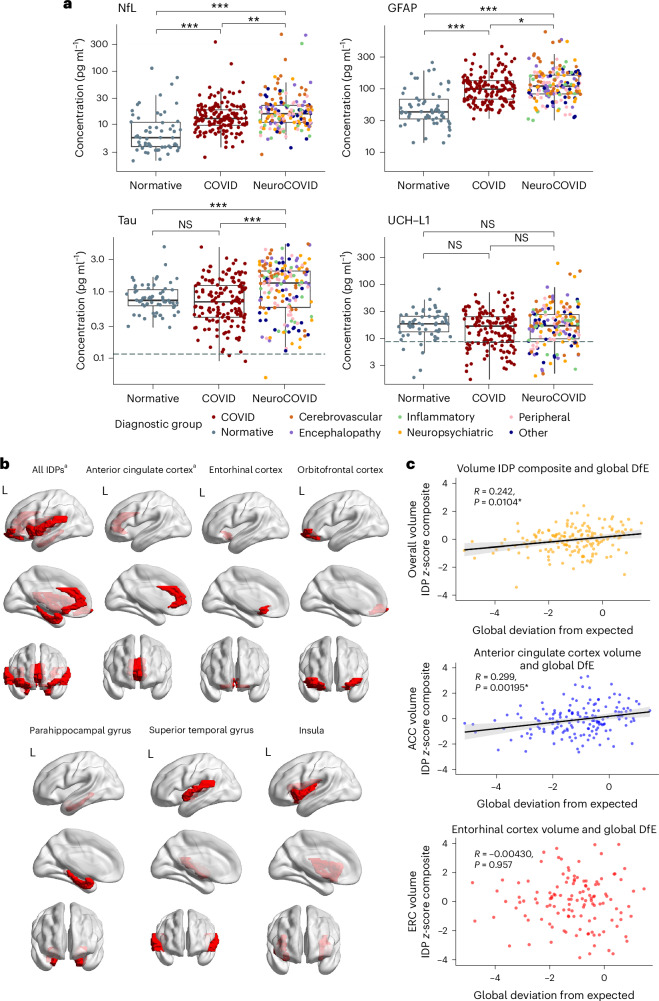
Brain injury biomarkers and neuroimaging. **a**, Brain injury markers (in pg ml^−1^) by diagnostic group. Lower limit of quantification (dashed line) if included in scale. Normative values from *n* = 60 healthy controls. *P < 0.05, **P < 0.01, ***P < 0.001; NS, nonsignificant, unadjusted two-sided Mann–Whitney *U*. **b**, Brain regions represented by the IDPs utilized in analyses. These regions are parcellated as per the Desikan-Killiany cortical atlas. For each region and regions combined, IDP composites for thickness and volume were utilized. ^a^IDP composites that have significant correlations with overall cognition (Supplementary Table 2). Created using Matlab and BrainNet Viewer^54^. **c**, Scatter plots for IDP composite *z*-scores against GDfE in the overall cohort, using Pearson’s correlation. The black line indicates the line of best fit (least squares method) and gray error band indicates the 95% CI. Significance persisting after adjusted for multiple comparisons based on the FDR approach (adjusted for *n* = 14 for each IDP composite test). Center line, median; box limits, upper and lower quartiles; whiskers, 1.5× IQR. ACC, anterior cingulate cortex; ERC, entorhinal cortex; UCH-L1, ubiquitin carboxy-terminal hydrolase L1.

Median (IQR) 384 (155–574) days after COVID-19, median (IQR) serum neurofilament light chain (NfL, a marker of axonal injury), and glial fibrillary acidic protein (GFAP; a marker of astrocyte injury) were significantly raised in patients who had had COVID-19 compared with healthy controls (healthy control versus COVID: NfL 5.46 (3.66–10.5) versus 12.4 (9.2–18.0) pg ml^−1^ (*U* = 2,151, estimate (CI) = 5.84 (4.09 to 7.54), effect size = 0.41, *P* < 0.001) and GFAP 42.4 (33.3–69.6) versus 94.3 (65.6–128.2) pg ml^−1^ (*U* = 1,824, estimate (CI) = 43.4 (31.2 to 56.8), effect size = 0.46, *P* < 0.001)). NfL and GFAP were further raised in those with neurological complications (COVID versus NeuroCOVID: NfL 12.4 (9.2–18.0) versus 15.2 (10.5–21.7) pg ml^−1^ (*U* = 10,234, estimate (CI) = 2.64 (4.33 to 1.07), effect size = 0.18, *P* = 0.001) and GFAP 94.3 (65.6–128.2) versus 105.4 (79.9–154.8) pg ml^−1^ (U = 11,246, estimate (CI) = 12.3 (23.6 to 0.685), effect size = 0.12, *P* = 0.039)) (Fig. 3a). Tau was raised exclusively in those with neurological complications (COVID versus NeuroCOVID: 0.69 (0.40–1.22) versus 1.32 (0.57–1.98) pg ml^−1^ (*U* = 8,854, estimate (CI) = 0.452 (0.260 to 0.661), effect size = 0.27, *P* < 0.001)).

#### Hypothesis 5: structural volumetric changes on MRI

Participants who underwent neuroimaging in the NeuroCOVID (*n* = 84 of 190) and COVID (*n* = 73 of 161) groups were similar to the overall cohort in median (IQR) age (52 (44–60) and 51 (45–57) years) and proportion of males (60 of 84 (71%) and 45 of 73 (62%)). The thickness and volume of regions represented by the composite image-derived phenotype (IDP) *z*-scores did not differ significantly between NeuroCOVID and COVID groups (Fig. 3b and Extended Data Table 4). One-way analysis of variance revealed a significant difference in IDP composites between diagnostic subgroups in terms of global thickness composite (*F* = 3.223, *P* = 0.00524) but this did not persist after false discovery rate (FDR) correction (*P* = 0.0734). Post hoc Tukey group comparisons for this thickness composite found significant differences between the neuropsychiatric subgroup and three subgroups: cerebrovascular (mean difference = 0.871, adjusted *P* = 0.0251), encephalopathy and/or delirium (mean difference = 0.936, adjusted *P* = 0.0119) and peripheral (mean difference = 0.769, adjusted *P* = 0.0395).

Pearson’s correlations between GDfE scores and IDP composites indicated significant correlations between overall cognition and the total brain IDP composite in the NeuroCOVID group (*R* = 0.296, *P* = 0.0444) and the overall cohort (*R* = 0.272, *P* = 0.0041; Extended Data Table 2). Global volume composite had significant correlations with cognitive deficits in the overall cohort (*R* = 0.242, *P* = 0.0022) (Fig. 3c), with a correlation in the NeuroCOVID group (*R* = 0.271, *P* = 0.0127) but not persisting after FDR correction. The bilateral volume of anterior cingulate cortex was significantly and moderately positively correlated with overall cognition in the NeuroCOVID group (*R* = 0.307, *P* = 0.0444), the COVID group (*R* = 0.307, *P* = 0.0280) and the overall cohort (*R* = 0.299, *P* = 0.00195; Fig. 3c).

#### Cluster analysis and multifaceted models

**Fig. 4:**
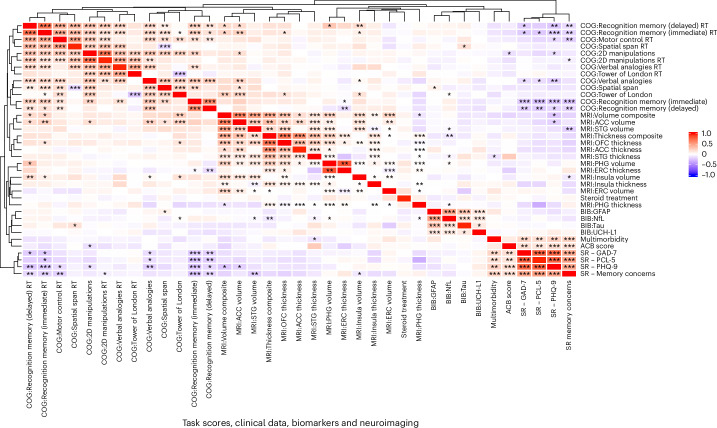
Heatmap and unsupervised cluster analysis. Heatmap and unsupervised cluster analysis (Euclidean, complete) in the full cohort (*n* = 351) of cognitive tasks shaded by correlation (Spearman), including cognition (accuracy and inverse RT), clinical variables, biomarkers and neuroimaging. *P < 0.05, **P < 0.01, ***P < 0.001 two-tailed Spearman correlation adjusted for multiple comparisons (37 ×3 7 matrix, *n* = 1,369) using the FDR approach. ACB, anticholinergic burden; BIB, brain injury marker; COG, cognitive task; OFC, orbitofrontal cortex; PHG, parahippocampal gyrus; SR, self-report; STG, superior temporal gyrus.

An unsupervised cluster analysis demonstrated that faster RT in memory tasks correlated with parahippocampal gyrus, anterior cingulate cortex and insula volumes (Fig. 4). Insula volume (*r* = 0.15) and orbitofrontal cortex thickness (*r* = 0.14) were correlated with executive function. Symptoms of depression were negatively correlated with immediate memory (*r* = −0.25), language (*r* = −0.20) and perceptual–motor function (two-dimensional manipulations *r* = −0.12) as well as anterior cingulate cortex volume (*r* = −0.20). Subjective memory impairment was associated with inaccurate (*r* = −0.24) and slow (*r* = −0.19) responses on memory tasks and reduced superior temporal gyrus (*r* = −0.20) and insula (*r* = −0.091) volume. Raised NfL in serum was weakly correlated with reduced thickness composite (*r* = −0.102) and reduced superior temporal gyrus volume (*r* = −0.033) and thickness (*r* = −0.048).

The NeuroCOVID multifaceted model contained 89% of individual data points, the rates of missingness for additional variables were anterior cingulate cortex volume (56%), GFAP (9%) and NfL (9%). The NeuroCOVID multifaceted model (*n* = 54 of 190, *R*^2^ = 0.68, *P* = <0.001) (Table 2), demonstrated cognitive deficits were associated with age (coefficient (s.e.) = −0.044 (0.017), *P* = 0.011), multimorbidity (0.31 (0.13), *P* = 0.025) and anterior cingulate cortex volume (0.23 (0.091), *P* = 0.017). The COVID multifaceted model contained 90% of individual data points, the rates of missingness for additional variables were anterior cingulate cortex volume (55%), GFAP (7%) and NfL (7%). In the COVID group (*n* = 53 of 161, *R*^2^ = 0.68, *P* < 0.001), cognitive deficits were associated with symptoms of depression (−0.056 (0.028), *P* = 0.050), increased multimorbidity (−0.20 (0.083), *P* = 0.021) and a raised GFAP (−0.0081 (0.0032), *P* = 0.017). Days since COVID-19 illness was not significantly associated (coefficient (s.e.)) with GDfE in the NeuroCOVID (0.000099 (0.0020)) or COVID group (0.0018 (0.0020)).

## Discussion

This prospective, national, multicenter study of 351 COVID-19 patients who required hospitalization with and without new neurological complications demonstrated that postacute cognitive deficits, relative to 2,927 matched controls, were associated with elevated brain injury markers in serum and reduced gray matter volume. In contrast to studies early in the pandemic that identified dysexecutive syndromes predominant in acute infection^15,18^, our study found global, persistent cognitive deficits even in those hospitalized without clinical neurological complications. When compared with normative age-matched data, these deficits were equivalent in magnitude to aging from 50 to 70 years of age^1^. This study indicated cognitive deficits were associated with the severity of the initial infective insult, postacute mental health status and a history of COVID-19 associated encephalopathy, with strong concordance between subjective and objective deficits. Despite some improvement at the first follow-up, by the second there was a plateau in the cognitive recovery trajectory. In addition, there was evidence of ongoing neuronal and astrocytic injury 1 year after acute COVID-19, even in those without neurological complications, with demonstration of underpinning neuroanatomical substrates as seen in other studies^30–33^.

The findings are both clinically relevant and biologically plausible. Raised brain injury markers have been demonstrated in acute and postacute COVID-19 and are associated with dysregulated innate and adaptive immune responses^21,34^. Similarly, meta-analysis has demonstrated higher NfL and GFAP in COVID-19 patients compared with healthy controls, and an association between these serum brain injury markers and COVID-19 severity and poorer outcomes^35^. The pattern of acute inflammatory proteins can predict postacute cognitive outcomes^36^. We have additionally shown that persistently raised serum GFAP was associated with postacute cognitive impairment. GFAP is expressed by astrocytes, which participate in neuroimmune interactions in the brain. Its appearance in the plasma typically indicates injury to these cells and it has been proposed as a prognostic biomarker for cognitive decline in the general population^37^.

Cognitive deficits were global, of substantial magnitude and spanned both accuracy and RT, echoing the findings of a recent study which demonstrated that patients hospitalized with COVID-19 had a broader cognitive deficit profile than those not hospitalized^38^. Future work should explore whether the cognitive deficits reported in community cohorts represent a continuum to this posthospitalized cohort, or whether additional mechanisms drive persistent deficits in those with a history of severe acute illness. Deficits were moderately to strongly associated with symptoms of depression, and the anterior cingulate cortex volume, which has functional roles in connecting cognition, attention and emotion^39^. An attentional basis for cognitive impairment with associated difficulties in memory encoding would be consistent with the global nature of the deficits including the immediate memory task. The anterior cingulate cortex is also frequently implicated in studies of depression utilizing positron emission tomography targeting translocator protein, which is interpreted as indicating microglial activation or neuroinflammation^40^. Longitudinal research using UK Biobank data reported volume loss in the anterior cingulate cortex and other limbic structures following mild SARS-CoV-2 infection^14^, but previous literature has also shown that the anterior cingulate cortex has reduced volume in older age^41,42^. Other studies have demonstrated reduced gray matter volume in cortical, limbic and cerebellar areas in post-COVID patients when compared with healthy controls^43^. This gray matter volume loss was correlated with white matter axial and mean diffusivity, as well as significantly associated with cognitive dysfunction relative to healthy controls. These cognitive and neuroimaging alterations have been identified as being greater in those patients who were hospitalized than in those who were not^43^. In our unsupervised cluster analysis, reduced cortical thickness, particularly in the superior temporal gyrus, was found to be associated with raised NfL, potentially indicating a regional substrate for axonal injury in this population. Some literature has suggested that neuroinflammation and neurodegeneration can mediate structural brain changes and neuropsychiatric sequelae^44,45^, and that serum NfL might be associated with changes to the superior temporal gyrus in these contexts^46^. The severe persistent deficits observed in those with COVID-associated acute encephalopathy, who did not have a pre-COVID history of neurological disease, suggest that a picture of encephalopathy and/or delirium in the context of infection is not just an unmasking of latent cognitive impairment but rather may precede lasting brain dysfunction^6^.

Advancing mechanistic understanding of post-COVID cognitive deficits has the potential to provide insight into therapeutic targets. This analysis implicates neurochemical and neuromodulatory mechanisms that both have potential to be targeted. There is growing biochemical evidence that neurological complications in COVID-19, including cognitive impairment, are immune-mediated^47^. If the anterior cingulate cortex were confirmed to be a nexus of late deficits in the postacute phase, its dopaminergic neurochemical linkage could provide a target for neuromodulatory therapy, with potential for utilizing drugs already approved for use in humans, as well as attention training therapies^48^.

The strengths of this study included its multimodality such as the use of robust longitudinal cognitive assessment, high-quality clinical data, serum biomarkers and nationally harmonized three Tesla neuroimaging data. Importantly, the GDfE scores reported represent how cognitive performance differs from what would be expected on an individual level based upon age, sex, level of education and first language, using data from a large normative dataset^2^. This reduces the risk of confounding due to premorbid state. It is possible that additional variance may be accounted for by elements such as socioeconomic factors and comorbidities. However, the matching of controls for the key factors that are established to affect cognitive performance on Cognitron testing, most importantly age and education, minimizes the potential for confounding^1^. Previous research has demonstrated that the pandemic context itself affected cognitive decline, but the effect sizes were too small to explain the deficits observed in this study^49^. The inclusion of patients with neurological complications allowed more complete assessment of the heterogeneous impact of COVID-19 on brain dysfunction. Although the method of case identification may have varied by site according to local clinical services, inclusion criteria for neurological complications were standardized nationally, and based on pre-published clinical case definitions^50^ with a bi-weekly clinical case evaluation panel, to ensure consistency. Nevertheless, to account for any potential regional effects, these were accounted for by inclusion of study recruitment region in regression models. The preregistered statistical analysis plan was conducted with minimal deviation and provides increased confidence in the results, which were broadly consistent with documented hypotheses. Limitations included the lack of premorbid assessment or acute biomarkers beyond routine clinical tests, the earlier assessment of NeuroCOVID patients and probable age- and severity-selection bias in those completing study assessments, particularly computerized cognitive assessment and MRI scanning. Although there was a significant difference between the COVID and NeuroCOVID groups in terms of days between COVID-19 and assessment, there was no significant correlation between days since COVID-19 and cognitive outcome in either group. The lack of genetic sequencing data for SARS-CoV-2 variants means the impact of admission epoch and viral clade on outcomes can only be assessed approximately. Complete case analysis can introduce bias, the extent of which depends upon the pattern of missingness and whether missingness is at random. We assumed data were missing at random. PHQ-9 was the only patient-reported value included in modeling with missingness (9% in NeuroCOVID and 11% in COVID), which could potentially introduce bias because it is possible that the underlying value is related to the missingness. The sample size for multifaceted models was limited by the number of patients with neuroimaging. There is the possibility of residual confounding when applying normative models, and the observational nature of the study, in particular the lack of preinfection data, means that pre–post infection change and causality cannot be inferred. It is important to note that the normative sample recruitment partially overlapped with the pandemic period. Individuals were specifically asked whether they had suspected or confirmed COVID-19 at the time of, or before, cognitive testing, and were excluded from the normative sample if this was the case. However, it is likely that some participants in the normative sample may have had asymptomatic infections given the population size. In addition, the normative control group were not matched for comorbidities, vaccination status or socioeconomic status beyond level of education.

The neuroimaging analysis exclusively uses preselected brain regions and the UK Biobank pipeline does not completely address some potential confounds such as head motion. Although structural scans, as utilized in this study, are not thought to suffer from degradation of image quality as a result of head motion to the same extent as other modalities, it is worth acknowledging that such confounds could increase the risk of false positives^51,52^. However, this study aimed to address this by excluding scans with marked motion artifact^53^. The region of interest-based neuroimaging analysis raises important candidate regions potentially underpinning the cognitive deficits seen, but reduces this study’s ability to identify unexpected regions’ involvement in such deficits, and might underestimate the importance of nonspecified regions. Similarly, current pipeline approaches limit the analysis of certain brain regions, such as the brainstem or basal ganglia. As such, future COVID-CNS neuroimaging analyses might utilize voxel-based or whole-brain approaches to more deeply characterize the nature of structural change in the brain post-COVID, and identify further brain areas relevant to cognitive impairment in this context. Finally, the analysis of recovery trajectories was underpowered, which limits interpretation, but there was evidence of a trend toward recovery that continued into the second year.

Taken together, this prospective multicenter longitudinal cohort study of patients hospitalized with COVID-19 illness found evidence of pervasive global cognitive impairment, associated with persistently raised brain injury markers, depression symptomatology and reduced anterior cingulate cortex volume. A strong concordance between subjective and objective cognitive deficits, underpinned by neuroanatomical and biochemical changes at almost 1 year postinfection, indicates that patient experience needs to be acknowledged by clinicians in this context. However, care needs to be taken in both inferring cause and effect, and extrapolating these results to a broader COVID-19 population. Mechanisms underpinning this potentially immune-mediated construct of depression, cognition and brain injury need to be further elucidated to allow the development of targeted therapeutic interventions.

## Methods

### Study population

Patients aged ≥16 years were recruited over 19 months (March 2021 to October 2022) from 17 UK sites through the COVID-CNS, a case-control study within the National Institute of Health Research (NIHR) COVID-19 BioResource. Either the participant or their next-of-kin provided informed consent (REC reference 17/EE/0025; 22/EE/0230 (East of England—Cambridge Central Research Ethics Committee)). COVID-CNS included hospitalized patients with COVID-19 without a previous relevant neurological diagnosis, who have had a new acute neurological or psychiatric complication (NeuroCOVID) alongside COVID-19 controls without these diagnoses (COVID). NeuroCOVID patients were recruited if they met the previously published study-wide case definitions and the inclusion and exclusion criteria detailed in Supplementary Table 1 (ref. ^50^). The NeuroCOVID group were identified by referral or admission to neurology, or by notification to the study team by the responsible clinician. Sites additionally screened relevant lists, for example, using clinical coding. The COVID group were recruited to match the NeuroCOVID group, matched on a group level by age, sex, ethnicity, pre-COVID clinical frailty status, COVID-19 severity and epoch of admission during the pandemic^55,56^. Sex was self-reported. Admission dates were categorized into 6-month blocks as per input from the Infectious Diseases Experts at the National Medical Research Council Clinical Trials Unit and multidisciplinary Clinical Case Evaluation Panel, to reflect phases of the UK epidemic dominated by circulation of different SARS-CoV-2 variants, and changes in clinical practice^57^. Some neurological or psychiatric complications required secondary care input without hospitalization, partially related to pandemic pressures and risk assessments, and a proportion of the COVID group were therefore recruited who attended the emergency department but were not admitted. COVID-19 was defined by the WHO COVID-19 case definition^58^.

This analysis contains a patient subset that completed cognitive testing (Fig. 1). Participants were assessed at a single postacute appointment (1–26 months after discharge), in which all assessments were undertaken including a computerized cognitive assessment (Cognitron), patient-reported measures, blood sampling for brain injury markers, 3 T MRI and a clinical examination. Self-reported measures included PCL-5, GAD-7, PHQ-9 and Chalder Fatigue Scale. Multimorbidity, defined as two or more comorbidities and anticholinergic burden score (a measure of how many medications taken might cumulatively contribute to an anticholinergic effect) were collected from past medical history and medications reflecting the admission timepoint^59^. To create a normative community comparator group, we subsampled individuals from a large dataset of cognitive assessments completed on a population volunteer sample between December 2019 and May 2020. For each COVID-CNS participant we subsampled approximately eight volunteers matched for age, sex, first language and level of education, resulting in a community comparator group of *n* = 2,927 in total^1,2^. These individuals had not tested positive for COVID-19, and reported that they did not suspect having had COVID-19 at the testing timepoint, although the possibility of asymptomatic infection cannot be excluded. The research team completed a standardized case record form using ‘Qualtrics’, to collect harmonized clinical data across sites regarding acute admission and neurological complications.

### Eligibility criteria

Patients with pre-existing neurological or psychiatric disorders managed in secondary care or pre-existing cognitive impairment were excluded. To ensure consistency nationwide, if there was doubt about the eligibility of a potential case identified by a recruiting team, this was discussed at the national multidisciplinary case evaluation panel.

### Cognitive outcome

The cognitive assessment included seven tasks from the Cognitron assessment battery completed once under supervised conditions and twice online during follow-up (details of the tasks are given Supplementary Note 1). We included patients within the COVID-CNS cohort who had completed at least the first supervised assessment. Cognitron is sensitive, specific and valid in the general population and disease cohorts^1,2,60,61^. Cognitive tasks were selected to sample across five domains defined by the DSM-5 classification:^62^ executive function, learning and memory, complex attention, perceptual–motor control and language. Accuracy and median RT values were extracted by task, comprising 13 measures. These data were transformed into Deviation from Expected (DfE) scores using established linear models trained on a large normative dataset (>400,000 individuals) designed to predict performance based upon demographics. In this analysis, GDfE, DfE accuracy and DfE RT represent how an individual performs compared with what would be expected based upon their age, sex, first language and level of education. Any cognitive impairment was defined as GDfE less than expected (<0). A technical correction was applied excluding those responding unfeasibly fast or slow based upon normative data. Follow-up 1 and follow-up 2 were completed 3 and 6 months following the postacute assessment. Recovery of cognitive performance was calculated as GDfE at follow-up 1 minus GDfE at postacute appointment.

Subjective cognitive impairment was assessed by a binary question, ‘Are you concerned about your memory, because it affects how you work or the way you live from day to day?’.

### Brain injury marker measurement

Brain injury markers were measured in serum using a Quanterix Simoa kit run on an SR-X Analyzer (Neurology 4-Plex A Advantage Kit; Quanterix, cat. no. 102153). We assayed NfL, ubiquitin carboxy-terminal hydrolase L1, tau and GFAP. Normative data were measured in stored serum samples from *n* = 60 healthy controls recruited to the NIHR BioResource ‘general population cohort’ before the COVID-19 pandemic. These persons were chosen to be representative for the main variable associated with normative brain injury biomarker levels (age) relative to the COVID-CNS cohort. The median (IQR, range) age was 50 (20–79) years and sex distribution was also representative of the COVID-CNS cohort^34^.

### Neuroimaging

This study utilized a published standardized protocol, with harmonized MRI scans across multiple sites, which demonstrated very good reliability between sites through a ‘traveling heads study’^53^. This protocol utilizes the existing UK Biobank IDP MRI analysis pipeline^51–53,63^. As part of structural imaging processing, this pipeline includes removal of face, brain extraction and registration to the MNI152 brain template, maximizing comparability of scans^52,64^. Field map correction was performed and FAST used to segment tissues into gray matter, white matter and cerebrospinal fluid^53,65^. SIENAX analysis then estimated volume measures, utilizing surface of skull to normalize brain tissue volumes for head size (compared with the MNI152 template)^53,66^. From these measures specific brain regions were selected based on extant literature a priori to analysis: the parahippocampal gyrus, entorhinal cortex, orbitofrontal cortex, anterior cingulate cortex, insula and superior temporal gyrus^14,67–71^. MRI data were processed with FSL and Freesurfer, using the established UK Biobank pipeline^51,53,63^, modified for COVID-CNS, to produce biologically relevant metrics of brain structure and function—IDPs. IDPs from T1- and T2-FLAIR-weighted MRI were obtained for global brain regions and for cortical regions as defined by Desikan-Killiany parcellation. IDPs represent gray matter thickness, volume and surface area. Fifty-four of these IDPs were selected as representative of general brain structure and the a priori selected brain regions. Volume and surface IDPs were found to be collinear (variance inflation factor >10) and so 38 IDPs representing volume and thickness were included in subsequent analysis (for a full list, see Supplementary Table 2). Individual IDPs were compared with the COVID-CNS population means and standard deviations to calculate *z*-scores. Available *z*-scores for each region—for example, right and left hemisphere anterior cingulate cortex volume—were combined to produce a composite *z*-score for each of the six prespecified regions. The authors also aimed to summarize IDP variance across disparate regions into single measures, so combined all relevant regional IDPs to produce further composites: volume IDP *z*-scores for a volume composite, thickness IDP *z*-scores for a thickness composite and all IDP *z*-scores for a total composite.

### Model development

Candidate variables for linear models were predefined in the statistical analysis plan. Models are presented separately in the NeuroCOVID and COVID groups and represent complete case analysis. Models were developed based upon a fixed set of modeling decisions (Supplementary Table 6). Clinical models contain the clinical variables in hypotheses 3 and 4: age, WHO COVID-19 disease severity, presence of an acute neurological or psychiatric complication, multimorbidity and mental health comorbidities, Rockwood Clinical Frailty Scale, level of education and previous treatment with dexamethasone during acute illness. Acute serum inflammatory markers were excluded owing to missingness >20%. Collinearity was assessed using correlation matrices (Supplementary Fig. 1). Fatigue^72^, subjective cognitive impairment and mental health measures were found to be collinear. PHQ-9 score was considered most clinically relevant based on existing literature and explained the most variance in GDfE and was therefore included in modeling^73^. Multifaceted models contain core clinical variables (PHQ-9, multimorbidity and clinical diagnostic subgroup) and, based on existing literature at the time of model development, NfL, GFAP and anterior cingulate cortex volume^14,35–37,41,42^. Date of admission, days since COVID-19 and recruitment site (grouped as ‘London’, ‘North’ and ‘South’) were included in clinical and multifaceted models with interaction term Admission date : Days since COVID-19. Because of sample size restrictions, recovery models included days since COVID-19, core clinical variables, NfL and GFAP. Within the preregistration, three sample size calculations were undertaken to determine adequate power (95%) at the 0.05 significance level for the cross-sectional analysis.

### Statistical analysis

The full analysis plan was preregistered before data access and is openly available via the Open Science Framework^28^. In summary, the primary outcome measure was GDfE on computerized cognitive assessment. DfE effect sizes are calculated comparing COVID-CNS participants with matched community controls. We used standard two-sided *P* < 0.05 criteria for determining statistical significance. Unsupervised hierarchical cluster analysis (Euclidean, complete) was undertaken to explore the correlations between cognitive scores, prespecified clinically important variables, brain injury markers and neuroimaging IDPs^74^. There were minor deviations from the analysis plan: there were seven individuals in the overall COVID-CNS cohort who had non-COVID respiratory illness and were excluded from this analysis owing to the small numbers. In addition, the community normative group was not stratified by COVID-19 status owing to a lack of data. We report multiple regression models for GDfE rather than accuracy and RT separately to improve clarity. We based models on complete case analysis rather than multiple imputation because existing data was deemed sufficient (<20% missingness). For MRI analysis, we report the analyses of a priori defined regions. Cortical volume and surface area were collinear and therefore cortical volume only was included (variance inflation factor >10). The statistical analysis plan was otherwise conducted as documented. Statistical analyses were performed in R (R Foundation, v.3.6.1 or later). Potential confounders were included as candidate variables in all multiple regression models. The GDfE score represents performance compared with what would be expected by age, sex, level of education and first language and therefore reduces the risk of confounding from these variables. GDfE is based on linear models trained on normative data from >400,000 individuals.

### Multiple comparisons

When appropriate, analyses utilized correction for multiple comparisons based on FDR methodology. FDR was applied with a threshold of 5%. Analyses which utilize FDR, the number of tests corrected for and the justification can be found in Supplementary Table 7.

### Reporting summary

Further information on research design is available in the Nature Portfolio Reporting Summary linked to this article.

## Online content

Any methods, additional references, Nature Portfolio reporting summaries, source data, extended data, supplementary information, acknowledgements, peer review information; details of author contributions and competing interests; and statements of data and code availability are available at 10.1038/s41591-024-03309-8.

## Supplementary information

Supplementary Information Supplementary Tables 1–9, Note 1 and Fig. 1.

Reporting Summary

## Figures and Tables

**Extended Data Table 1:**
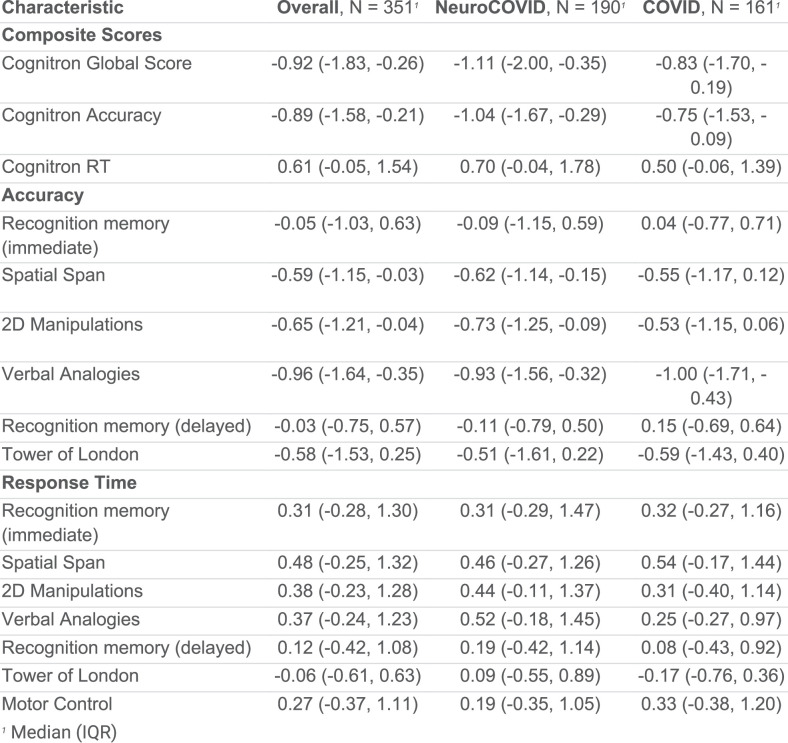
Composite and individual task Deviation from Expected Scores in NeuroCOVID and COVID groups

**Extended Data Table 2:**
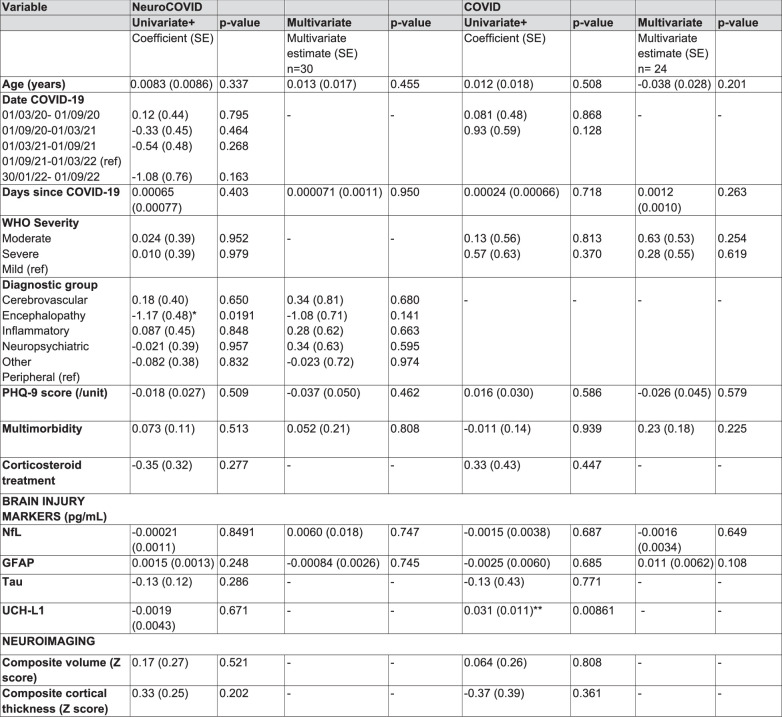
Factors associated with recovery in NeuroCOVID and COVID groups. Both models contain the interaction term Admission Date: Days since admission. Selected variables only are presented, - = not selected. Two-sided linear regression coefficient * p < 0.05, **p < 0.01, ***p < 0.001. +GDfE represents how an individual performs compared to what would be expected based upon their age, sex, first language and level of education

**Extended Data Table 3:**
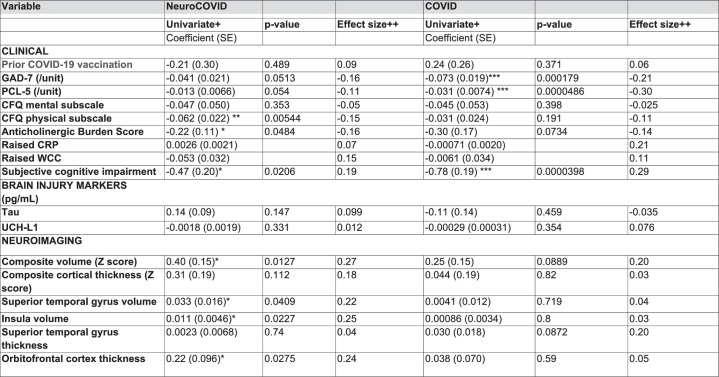
Univariate associations, clinical linear regression model and multifaceted linear regression models for Global DfE (GDfE) Score in NeuroCOVID and COVID groups. Pre-defined variables not included in multivariate analysis are described. Two-sided linear regression coefficient * p < 0.05, **p < 0.01, ***p < 0.001. +GDfE represents how an individual performs compared to what would be expected based upon their age, sex, first language and level of education. ++ Mann-Whitney U r for categorical variables, Spearman rho for continuous variables, Pearson r for image derived phenotypes; >0.1 small effect size, >0.3 medium effect size

**Extended Data Table 4:**
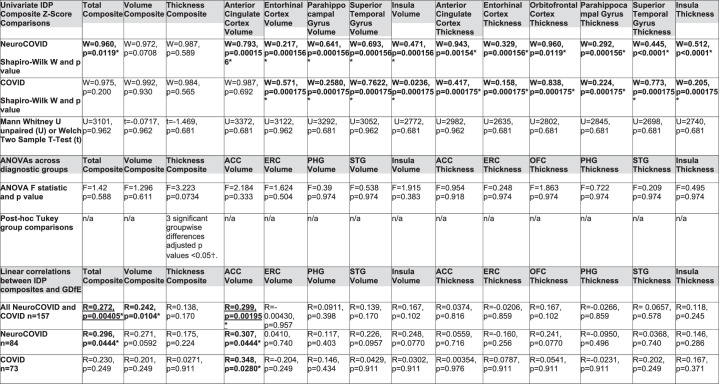
Univariate associations for composite IDP z-scores in NeuroCOVID and COVID groups, utilising two-sided Mann Whitney U unpaired if nonnormal distribution, or two-sided Welch Two Sample T-Test if normal distribution. Linear correlations between composite IDP z-scores and Global DfE (GDfE) Score in overall cohort, NeuroCOVID group and COVID group. ANOVA across COVID group and NeuroCOVID subgroups for composite IDP z-scores. All p-values adjusted using Benjamini-Hochberg procedure for False Discovery Rate (adjusted for n=14 for each IDP composite test, based on 14 tests in each group).♱The three significant groupwise differences found in post-hoc tukey group comparisons for the Thickness IDP composite were found between the Neuropsychiatric and Cerebrovascular subgroups (mean difference 0.871, adjusted p=0.0251), Neuropsychiatric and Encephalopathy and Delirium Group (mean difference 0.936, adjusted p=0.0119), and Neuropsychiatric and Peripheral subgroups (mean difference 0.769, adjusted p=0.0395)

## Data Availability

Individual-level data and samples from the COVID-Clinical Neuroscience Study are available for collaborative research by application through the NIHR BioResource Data Access Committee https://bioresource.nihr.ac.uk/using-our-bioresource/apply-for-bioresource-data-access/. The Committee decide on academic applications, with escalation to the NIHR BioResource Steering Committee for contentious applications, and/or applications from industry. Participants in the NIHR BioResource have all consented to the sharing of de-identified data with bona fide researchers worldwide, for research in the public interest. There are limits to these consents both by expectation and legal—some datasets may not be shared beyond a safe setting in the UK. The Data Access Committee aim to process data-only requests as quickly as possible and meet fortnightly to consider applications. Once approved, timeframes for data availability vary from 2 weeks to 6 months depending on the nature of the data requested.

## Code availability

Code is publicly available via https://github.com/tnggroup/covidcns.
