# Supplementary material for: Posthospitalization COVID-19 cognitive deficits at 1 year are global and associated with elevated brain injury markers and gray matter volume reduction
Source: Nat Med. 2024 Sep 23;31(1):245–57. doi: 10.1038/s41591-024-03309-8 (PMC11750706; doi:10.1038/s41591-024-03309-8)
Supplement: Supplementary file 1 — Supplementary Tables 1–9, Note 1 and Fig. 1. [file 41591_2024_3309_MOESM1_ESM.pdf]

nature medicine

Supplementary information

https://doi.org/10.1038/s41591-024-03309-8

Posthospitalization COVID-19 cognitive deficits at 1 year are global and associated with elevated brain injury markers and gray matter volume reduction

In the format provided by the authors and unedited

Supplementary Information

Supplementary Table 1: COVID-CNS inclusion and exclusion criteria

	INCLUSION	DEFINITION	CLINICAL CASE REVIEW PANEL	EXCLUSION
	GREEN	NEW ONSET		ALTERNATIVE CAUSE MORE LIKELY
Encephalopathy/encephalitis	Encephalopathy/Delirium	Altered Mental Status (AMS): Acute alteration in consciousnes, cognition, behaviour and/or personality (ideally with encephalopathic features on EEG) without other more specific diagnosis		Prior *diagnosed* cognitive impairment. Severe Hypoxia, Hypercapnoea, Sepsis, Iatrogenic or other primary non-CNS explaination
	Severe Encephalopathy	AMS with GCS ≤13 with/without seizures or movement disorder without evidence of CNS inflammation (as defined in encephalitis)		Alternative diagnosis more likely (e.g. illicit or iatrogenic drugs)
	Encephalitis	AMS with evidence of CNS inflammation: CSF pleocytosis and/or MRI inflammation and/or raised CSF protein and/or oligoclonal bands in CSF and/or recognised autoantibody in serum/CSF and/or EEG consistent with encephalitis.		Alternative infectious cause identified (e.g. HSV identified by PCR and/or intrathecal antibody synthesis)
Cerebrovascular	Ischaemic stroke	Rapidly developing clinical signs of focal (or global) disturbance of cerebral function, with symptoms, lasting ≥24 hours or leading to death, with no apparent cause other than vascular origin (ideally with CT/MRI evidence of infarct)		Vascular dementia, Previous stroke, Multiple vascular risk factors (Diabetes, hypertension, hypercholesterolaemia, smoking), Traumatic dissection
	Haemorrhagic stroke or other CNS haemorrhage	SAH or ICH identified on neuroimaging and/or xanthachromia in CSF		Anticoagulation, brain trauma, known malignant hypertension
	Cerebral venous sinus thrombosis	Cerebral venous sinus thrombosis identified on CT or MR venography		Known hyper-coagulable state prior to COVID-19 or recognised cause identified
Demyelinating	Acute Diseminated Encephalo-Myelitis (ADEM)	Sensory and/or motor symptoms attributable to multiple CNS demyelinating lesions with MRI confirmation of demyelinating lesions disseminated in space in both the intracranial and spinal structures		McDonald criteria for MS or other diagnsosis more appropriate (e.g. Neuromyelitis optica)
	Clinically Isolated syndrome (CIS)	Sensory and/or motor symptoms attributable to multiple CNS demyelinating lesions with MRI confirmation of demyelinating lesions disseminated in space in the intracranial structures only		McDonald criteria for MS or other diagnsosis more appropriate (e.g. Neuromyelitis optica)
	Myelitis	Sensory and/or motor symptoms and signs attributable to a spinal cord lesion, with evidence of demyelating lesion(s) of the spinal cord		McDonald criteria for MS or other diagnsosis more appropriate (e.g. Neuromyelitis optica)
Brainstem/Peripheral	Cranial nerve palsy or Rhombencephalitis	Objective clinical sign of any cranial nerve palsy		Alternative cause
	Guillain Barré syndrome and variants	GBS, or Miller Fisher syndrome (including AMSAN, ASAN, AMAN)		Known chronic inflammatory demyelinating polyneuropathy or autoimmune vasculitis
	Peripheral nerve dysfunction	Sensory and/or motor symptoms and signs attributable to peripheral nerve pathology (large fibre or small fibre) ideally with electrophysiological evidence		ITU stay >4 weeks at the time of symptom/sign onset
	Myositis	Symptoms and signs attributable to muscle inflammation including elevation of peripheral muscle enzymes		Known aetiology e.g. iatrogenic, neuroleptic malignant syndrome, first degree family history
Movement Disorder/Other	Hyperkinetic movement disorder	Myoclonus, hyperekplexia, hemibalismus, tics, opsoclonus, other		Recognised structural, metabolic, or degenerative pathology
	Hypokinetic movement disorder	Parkinsonism (bradykinesia, rigidity, and rest tremor)		Drugs with extrapyramidal (parkinsonian) side effects
	Seizure	Witnessed seizure of any description (generalised convulsive or focal with/without impaired awareness); AND/OR electrographic seizures seen on EEG. Includes status epilepticus.		Known epileptic patient prior to COVID-19 diagnosis
Neuropsychiatric	Psychosis	hallucinations (all modalities) and/or delusions (in clear consciousness/without disorientation?confusion?) and/or thought disorder		Known psychosis prior to COVID-19 (concurrent delirium?)/clear other cause?
	Mania/hypomania	e.g. elevated mood, pressure of speech, sleep disturbance, irritability		Known Mania and/or bipolar disorder prior to COVID-19
	Catatonia/other psychomotor	e.g. psychomotor retardation(or); mannerisms / posturing; echophenomena		Known catatonia and/or severe depression prior to COVID-19
	Neurocognitive (dementia-like) syndrome	e.g. subacute dementia		Known cognitive impairment prior to COVID-19
	Other organic presentation			steroid induced
		e.g. New onset depression or anxiety disorder within 6 weeks of COVID-19 symptoms onset, where depression is moderate or severe on ICD criteria.	e.g. New onset depression or anxiety from 6 weeks to 3-6 months after COVID-19 symptoms onset.	
			Obsessive-compulsive syndrome (DSM5)	
			Impulse control syndrome (DSM5)	
			Any disorder occuring in the perinatal period (conception-<1yrold baby)	

Supplementary Note 1: COVID-CNS: Cognitive tasks

Included tasks:

1. Recognition memory (immediate)
2. Spatial Span
3. 2D Manipulations
4. Verbal Analogies
5. Recognition memory (delayed)
6. Tower of London
7. Motor control

Tasks 1 and 5: Recognition memory

Participants memorise a word list at the start of the battery. They are then tested on immediate recall and delayed recall. This type of task is the mainstay of neuropsychological assessment of memory function. The Cognitron version has been validated with data from ~100,000 members of the general public.

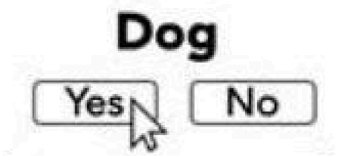

Task 2: Spatial span

The Spatial Span test measures spatial short-term memory capacity. It is a variant on the classic Corsi Block Tapping Test (Corsi, 1972). The participant is presented with a 4 x 4 grid, onto which is displayed a sequence of squares in different positions in the grid. The participant must then click the squares in the order that they were highlighted. The difficulty is incremented using a ratchet system, every time a sequence is recalled correctly, the length of the subsequent sequence is incremented by one. The test is terminated when three consecutive mistakes are made on a particular sequence length. The outcome measure is the maximum sequence length correctly recalled. Minimum level = 2, maximum level = 16, ISI = 0ms, encoding time = 1500ms. Population mean = 6.10, SD = 1.23.

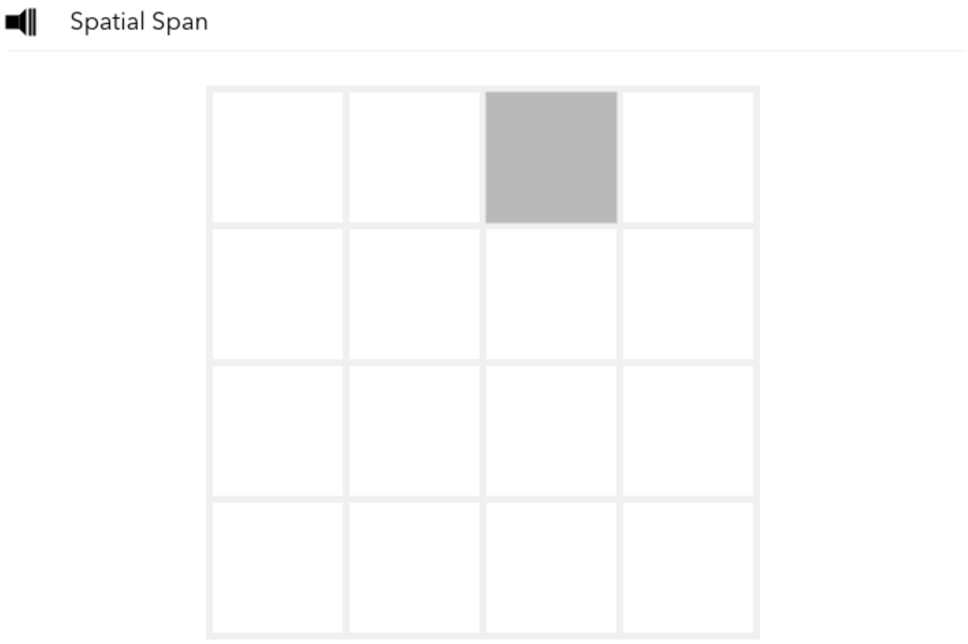

Task 3: 2D Manipulations

The 2D Mental rotation test measures the ability to spatially manipulate objects in mind (Silverman et al., 2000). In this version of the test, a grid with coloured squares is presented at the top of the screen, with a further four grids with coloured squares presented below (i.e. probe grids). One of the four grids is identical to the target grid above but is rotated by either 90, 180 or 270 degrees whilst the other grids differ by five squares. To obtain maximum points, the participant must indicate which of the four grids is identical to target, solving as many problems as possible within three minutes. For every correct response, the total score increases by one. The outcome measure is the total score. Population mean = 26.8, SD = 8.35.

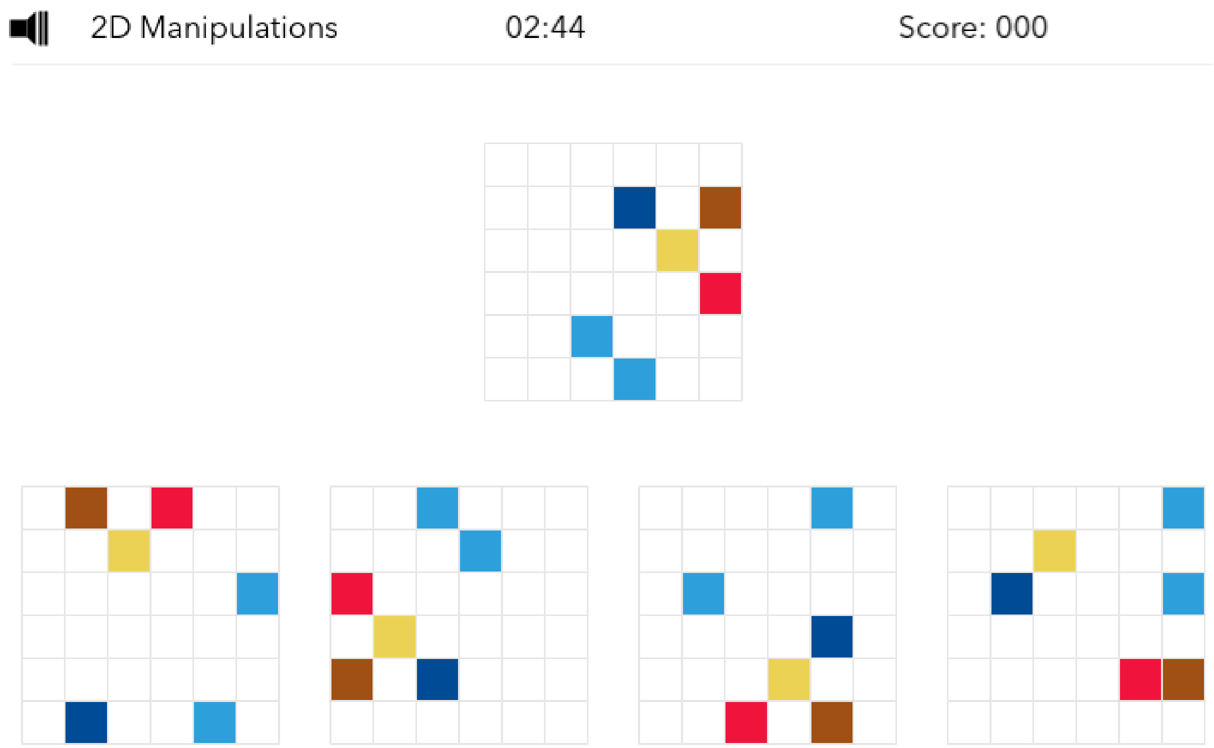

Task 4: Verbal Analogies

The Analogical Reasoning test measures semantic reasoning abilities. In this version of the test, participants are presented with two written relationships that they must decide have the same type of association or not (e.g. “Lion is to feline as cabbage is to vegetable”). Participants must indicate their decision by selecting the True or False buttons presented below the written analogies. Analogies are varied across semantic distance to modulate difficulty and associations types switch throughout the sequence of trials. To obtain maximum points, participants must solve as many problems as possible within three minutes. For every correct response, the total score increases by one. For every incorrect response, the total score decreases by one. The outcome measure is the total score. Population mean = 24.1, SD = 11.5.

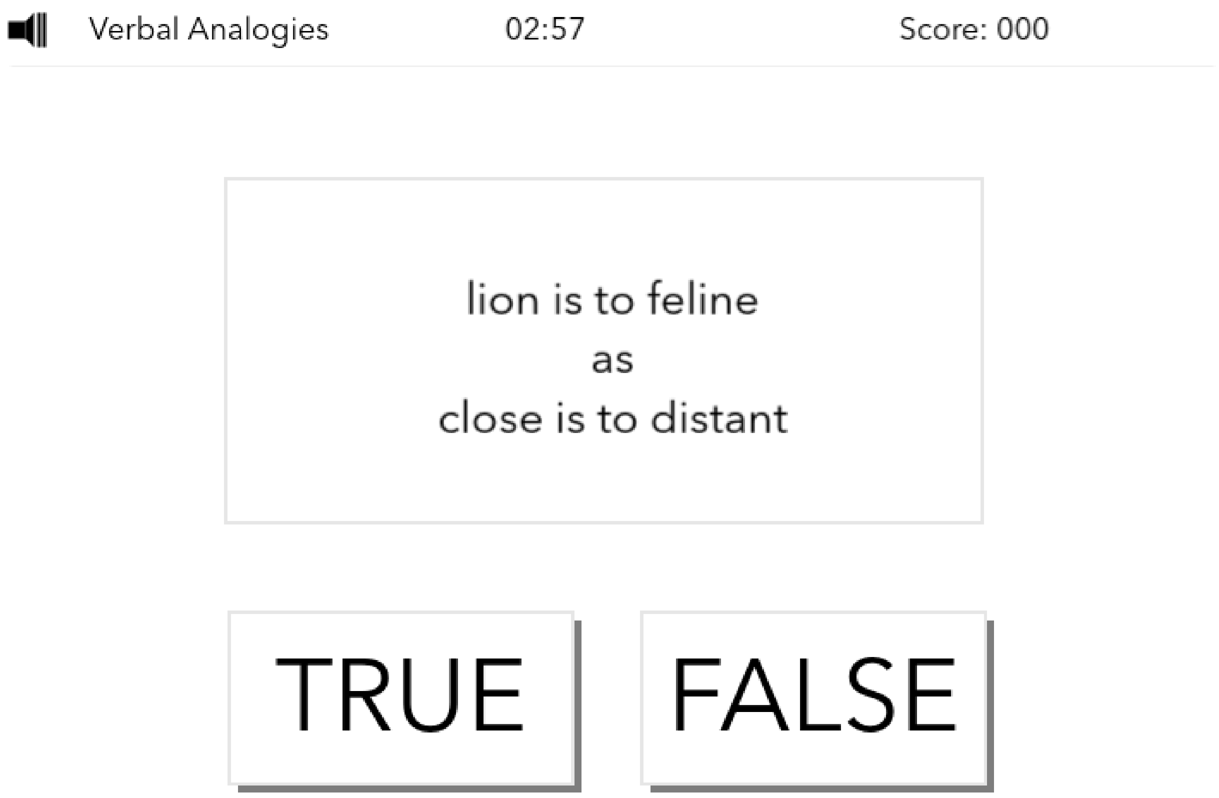

Task 6: Tower of London

The Tower of London test measures spatial planning. It is a variant on the original Tower of London Test (Shallice, 1982). The participant is shown two sets of three prongs with coloured beads on them. The first set is the initial state and the second set is the target state. The participant must work out the lowest number of moves it would take to transition from the initial state to the target state. They must then input this number using an on-screen number pad. This differs from the original test in that the participant is not allowed to move the beads, all calculation and planning must be done in their head. This is to prevent correct answers being reached through iterative error correction. The test consists of 10 trials of variable difficulty. The difficulty is scaled using the number of beads and the convolutedness, defined as the number of moves that must be made that do not place a bead in its final target position. The outcome measure is the total number of correct trials. Population mean = 6.57, SD = 2.62.

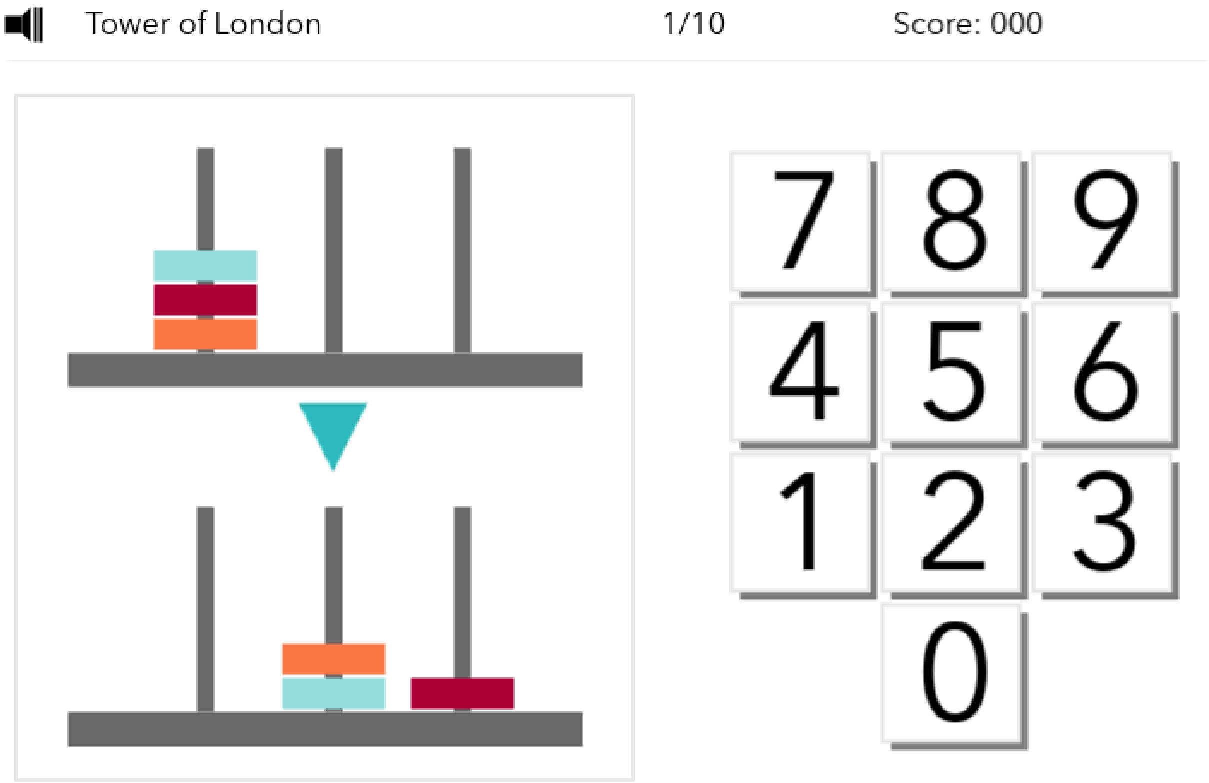

Task 7: Motor control

The motor control task assesses speed and therefore only response time is reported. Subsampled normative data were unavailable hence exclusion from Figure 2b.

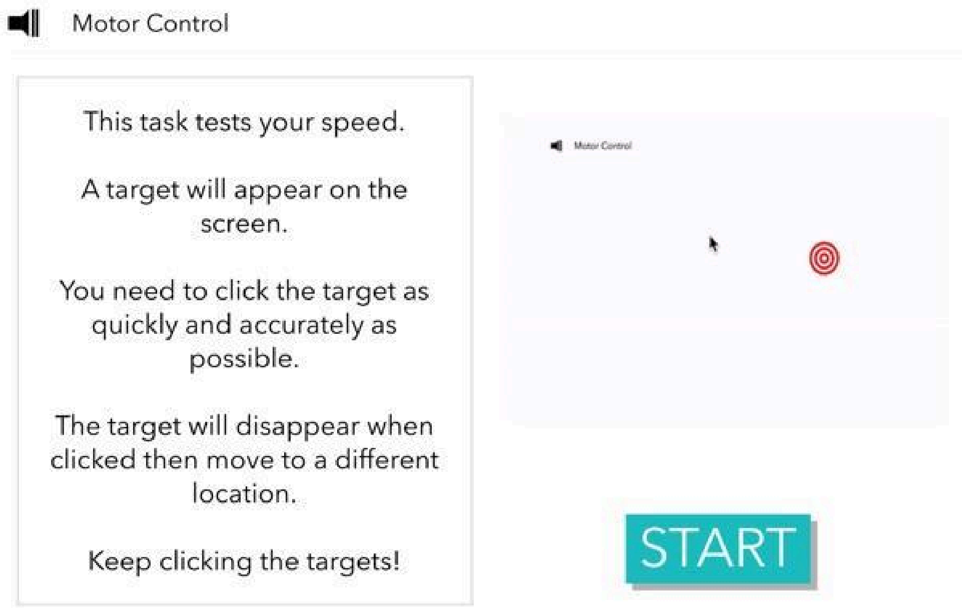

Supplementary Table 2: Image Derived Phenotypes (IDPs) considered for inclusion in analysis. All structural IDPs from Desikan-Killiany parcellation. Surface area IDPs were not used in final analysis due to collinearity (VIF >10) with volume IDPs.

Composite IDP				
Anterior Cingulate Cortex Volume	aparcdesikanlhvolume_caudalanteriorcingulate	aparcdesikanlhvolume_rostralanteriorcingulate	aparcdesikanrhvolume_caudalanteriorcingulate	aparcdesikanrhvolume_rostralanteriorcingulate
Entorhinal Cortex Volume	aparcdesikanlhvolume_entorhinal	aparcdesikanrhvolume_entorhina		
Orbitofrontal Cortex Volume	aparcdesikanlhvolume_lateralorbitofrontal	aparcdesikanlhvolume_medialorbitofrontal	aparcdesikanrhvolume_lateralorbitofrontal	aparcdesikanrhvolume_medialorbitofrontal
Parahippocampal Gyrus Volume	aparcdesikanlhvolume_parahippocampal	aparcdesikanrhvolume_parahippocampal		
Superior Temporal Gyrus Volume	aparcdesikanlhvolume_superiortemporal	aparcdesikanrhvolume_superiortemporal		
Insula Volume	aparcdesikanlhvolume_insula	aparcdesikanrhvolume_insula		
Anterior Cingulate Cortex Thickness	aparcdesikanlhthickness_caudalanteriorcingulate	aparcdesikanlhthickness_rostralanteriorcingulate	aparcdesikanrhthickness_caudalanteriorcingulate	aparcdesikanrhthickness_rostralanteriorcingulate
Entorhinal Cortex Thickness	aparcdesikanlhthickness_entorhinal	aparcdesikanrhthickness_entorhinal		
Orbitofrontal Cortex Thickness	aparcdesikanlhthickness_lateralorbitofrontal	aparcdesikanlhthickness_medialorbitofrontal	aparcdesikanrhthickness_lateralorbitofrontal	aparcdesikanrhthickness_medialorbitofrontal
Parahippocampal Gyrus Thickness	aparcdesikanlhthickness_parahippocampal	aparcdesikanrhthickness_parahippocampal		
Superior Temporal Gyrus Thickness	aparcdesikanlhthickness_superiortemporal	aparcdesikanrhthickness_superiortemporal		
Insula Thickness	aparcdesikanlhthickness_insula	aparcdesikanrhthickness_insula		
Anterior Cingulate Cortex Surface Area	aparcdesikanlharea_caudalanteriorcingulate	aparcdesikanlharea_rostralanteriorcingulate	aparcdesikanrharea_caudalanteriorcingulate	aparcdesikanrharea_rostralanteriorcingulate
Entorhinal Cortex Surface Area	aparcdesikanlharea_entorhinal	aparcdesikanrharea_entorhinal		
Orbitofrontal Cortex Surface Area	aparcdesikanlharea_lateralorbitofrontal	aparcdesikanlharea_medialorbitofrontal	aparcdesikanrharea_lateralorbitofrontal	aparcdesikanrharea_medialorbitofrontal
Parahippocampal Surface Area	aparcdesikanlharea_parahippocampal	aparcdesikanrharea_parahippocampal		
Superior Temporal Gyrus Surface Area	aparcdesikanlharea_superiortemporal	aparcdesikanrharea_superiortemporal		
Insula Surface Area	aparcdesikanlharea_insula	aparcdesikanrharea_insula		
Additional (Included in Volume and/or overall composites)	aseglhvolume_cortex	aparcdesikanlhthickness_GlobalMeanThickness	idpt1sienax_greynormalisedvolume	idpt1sienax_peripheralgreynormalisedvolume
	aseglhvolume_hippocampus	asegrhvolume_hippocampus		

Supplementary Table 3

Demographics of patients completing follow-up 1 (FU1), comparing NeuroCOVID and COVID groups. PAA= Post-acute assessment.

**Characteristic**	**Overall**, N = 81[1]	**NeuroCOVID**, N = 51[1]	**COVID**, N = 30[1]	**p-value**[2]
**Age**	55 (46, 63)	57 (46, 65)	53 (48, 60)	0.6
**Sex**				0.6
Female	30 (37%)	20 (39%)	10 (33%)	
Male	51 (63%)	31 (61%)	20 (67%)	
**First language**				0.3
English	70 (86%)	46 (90%)	24 (80%)	
other	11 (14%)	5 (9.8%)	6 (20%)	
**Level of education**				0.5
None of the above	5 (6.2%)	3 (5.9%)	2 (6.7%)	
College or university degree	37 (46%)	19 (37%)	18 (60%)	
A levels/AS levels or equivalent (School/vocational)	8 (9.9%)	7 (14%)	1 (3.3%)	
O levels/GCSEs or equivalent (School/vocational)	21 (26%)	15 (29%)	6 (20%)	
CSEs or equivalent (School/vocational)	3 (3.7%)	2 (3.9%)	1 (3.3%)	
NVQ or HND or HNC or equivalent (School/vocational)	6 (7.4%)	4 (7.8%)	2 (6.7%)	
Other professional qualifications (School/vocational)	1 (1.2%)	1 (2.0%)	0 (0%)	
**Clinical Frailty Scale**				0.7
Managing well	59 (86%)	36 (82%)	23 (92%)	
Mild	9 (13%)	7 (16%)	2 (8.0%)	
Moderate-severe	1 (1.4%)	1 (2.3%)	0 (0%)	
Unknown	12	7	5	
**WHO COVID-19 Severity**				0.063
Ambulatory mild disease	19 (29%)	14 (39%)	5 (17%)	
Hospitalised: moderate	28 (42%)	11 (31%)	17 (57%)	
Hospitalised: severe	19 (29%)	11 (31%)	8 (27%)	
Unknown	15	15	0	
**Admission date**				0.022
01/03/2020 – 01/09/2020	27 (38%)	13 (30%)	14 (50%)	
01/09/2020 – 01/03/2021	18 (25%)	12 (28%)	6 (21%)	
01/03/2021 – 01/09/2021	9 (13%)	9 (21%)	0 (0%)	
01/09/2021 – 01/03/2022	15 (21%)	7 (16%)	8 (29%)	
01/03/2022 - 01/09/2022	2 (2.8%)	2 (4.7%)	0 (0%)	
Unknown	10	8	2	
**Days since PAA**	111 (103, 163)	113 (103, 185)	111 (102, 122)	0.15
**PAA Cognitron Global Score**	-0.61 (-1.31, -0.12)	-0.61 (-1.35, -0.17)	-0.60 (-1.08, 0.07)	0.6
**PAA Cognitron Accuracy**	-0.57 (-1.08, 0.04)	-0.57 (-1.16, -0.04)	-0.52 (-1.03, 0.46)	0.2
**PAA Cognitron RT**	0.47 (-0.06, 1.19)	0.53 (-0.06, 1.18)	0.42 (0.05, 1.14)	0.9
**FU1 Cognitron Global Score**	-0.25 (-0.71, 0.29)	-0.30 (-0.71, 0.14)	-0.07 (-0.63, 0.36)	0.2
**FU1 Cognitron Accuracy**	-0.20 (-0.90, 0.56)	-0.48 (-0.94, 0.38)	0.10 (-0.81, 0.65)	0.2
**FU1 Cognitron RT**	0.01 (-0.36, 0.62)	0.09 (-0.22, 0.65)	-0.03 (-0.41, 0.31)	0.4

[1] Median (IQR); n (%)

[2] Two-sided Wilcoxon rank sum test; Pearson's Chi-squared test; Fisher's exact test

Supplementary Table 4

Demographics of patients completing follow-up 2 (FU2), comparing NeuroCOVID and COVID groups. PAA= Post-acute assessment

**Characteristic**	**Overall**, N = 59[1]	**NeuroCOVID**, N = 40[1]	**COVID**, N = 19[1]	**p-value**[2]
**Age**	56 (48, 65)	56 (48, 65)	55 (49, 63)	0.8
**Sex**				0.7
Female	26 (44%)	17 (43%)	9 (47%)	
Male	33 (56%)	23 (58%)	10 (53%)	
**First language**				0.4
English	53 (90%)	37 (93%)	16 (84%)	
other	6 (10%)	3 (7.5%)	3 (16%)	
**Level of education**				>0.9
None of the above	5 (8.5%)	3 (7.5%)	2 (11%)	
College or university degree	23 (39%)	14 (35%)	9 (47%)	
A levels/AS levels or equivalent (School/vocational)	4 (6.8%)	3 (7.5%)	1 (5.3%)	
O levels/GCSEs or equivalent (School/vocational)	15 (25%)	10 (25%)	5 (26%)	
CSEs or equivalent (School/vocational)	4 (6.8%)	3 (7.5%)	1 (5.3%)	
NVQ or HND or HNC or equivalent (School/vocational)	6 (10%)	5 (13%)	1 (5.3%)	
Other professional qualifications (School/vocational)	2 (3.4%)	2 (5.0%)	0 (0%)	
**Clinical Frailty Scale**				>0.9
Managing well	45 (88%)	32 (89%)	13 (87%)	
Mild	6 (12%)	4 (11%)	2 (13%)	
Unknown	8	4	4	
**WHO COVID-19 Severity**				0.006
Ambulatory mild disease	15 (29%)	13 (39%)	2 (11%)	
Hospitalised: moderate	22 (43%)	9 (27%)	13 (72%)	
Hospitalised: severe	14 (27%)	11 (33%)	3 (17%)	
Unknown	8	7	1	
**Admission date**				0.015
01/03/2020 – 01/09/2020	19 (35%)	10 (29%)	9 (47%)	
01/09/2020 – 01/03/2021	16 (30%)	14 (40%)	2 (11%)	
01/03/2021 – 01/09/2021	5 (9.3%)	5 (14%)	0 (0%)	
01/09/2021 – 01/03/2022	13 (24%)	6 (17%)	7 (37%)	
01/03/2022 - 01/09/2022	1 (1.9%)	0 (0%)	1 (5.3%)	
Unknown	5	5	0	
**Days since PAA**	203 (188, 251)	211 (196, 254)	189 (183, 207)	0.017
**PAA Cognitron Global Score**	-0.76 (-1.71, -0.33)	-0.94 (-1.98, -0.22)	-0.74 (-1.47, -0.57)	>0.9
**PAA Cognitron Accuracy**	-0.73 (-1.20, -0.15)	-0.78 (-1.32, -0.33)	-0.60 (-0.98, -0.15)	0.4
**PAA Cognitron RT**	0.71 (0.06, 1.54)	0.67 (-0.06, 1.58)	0.88 (0.27, 1.39)	0.9
**FU2 Cognitron Global Score**	-0.17 (-0.84, 0.34)	-0.18 (-1.21, 0.50)	-0.17 (-0.58, 0.23)	>0.9
**FU2 Cognitron Accuracy**	-0.25 (-1.01, 0.34)	-0.30 (-1.11, 0.38)	-0.18 (-0.54, 0.21)	0.7
**FU2 Cognitron RT**	0.13 (-0.45, 0.83)	0.20 (-0.48, 0.85)	0.06 (-0.36, 0.45)	0.9

[1] Median (IQR); n (%)

[2] Two-sided Wilcoxon rank sum test; Pearson’s Chi-squared test; Fisher’s exact test; Wilcoxon rank sum exact test

Supplementary Table 5: Secondary analysis. Univariate associations and effect sizes for Global DfE (GDfE) Score in the COVID group (n=161) and a randomly subsampled COVID group (n=66), matched to NeuroCOVID group for days since COVID-19. Pre-defined variables only are included, - = not included. - - - = no data. Two-sided linear regression coefficient * p < 0.05, **p < 0.01, ***p < 0.001. +GDfE represents how an individual performs compared to what would be expected based upon their age, sex, first language and level of education. ++ Mann-Whitney U r for categorical variables, Spearman rho for continuous variables, Pearson r for image derived phenotypes; >0.1 small effect size, >0.3 medium effect size.

Variable	COVID n=161			COVID subsample n=66		
	**Univariate+**	**Coefficient p-value**	**Effect size++**	**Univariate+**	**Coefficient p-value**	**Effect size++**
	Coefficient (SE)			Coefficient (SE)		
**CLINICAL PARAMETERS**						
**Age (years)**	-0.012 (0.0074)	0.117	-0.12	0.017 (0.012)	0.179	-0.12
**Level of education**						
Degree	0.75 (0.31)*	0.017	0.24	1.58 (0.52)**	0.00332	0.37
School, vocational	0.11 (0.32)	0.727	0.02	0.67 (0.52)	0.207	0.13
None of above (ref)						
**Clinical Frailty Scale**						
Mild (4-5)	-0.18 (0.56)	0.747	0.03	-0.91 (0.74)	0.225	0.16
Moderate-severe (6-8)	- - -	- - -	- - -	- - -		- - -
**Admission date**						
01/03/2020- 01/09/2020	-0.25 (0.26)	0.350	0.09	-0.30 (0.52)	0.566	0.13
01/09/2020-01/03/2021	-0.80 (0.26)**	0.00290	0.28	-0.71 (0.39)	0.0748	0.23
01/03/2021-01/09/2021	-0.47 (0.37)	0.210	0.15	-0.53 (0.52)	0.311	0.15
01/09/2021-01/03/2022 (ref)						
01/03/2022-01/09/2022	-0.18 (0.44)	0.689	0.04	1.24 (1.29)	0.340	0.30
**Days since COVID-19**	-0.00018 (0.00032)	0.574	-0.0099	-0.00057 (0.00066)	0.390	-0.12
**WHO COVID-19 Severity**						
Moderate	-0.81(0.28)**	0.00481	0.28	-1.05 (0.56)		0.28
Severe	-0.85(0.32)**	0.00807	0.34	-1.25 (0.60)*	0.042	0.51
Mild (ref)						
**PHQ-9 score (/unit)**	-0.065 (0.017)***	0.000192	-0.29	-0.077 (0.027)**	0.00663	-0.33
**Multimorbidity**	-0.16 (0.058)**	0.00734	-0.11	-0.21 (0.082)*	0.0146	-0.13
**Steroid treatment**	0.028 (0.20)	0.89	0.04	-0.13 (0.35)	0.708	0.04
**BRAIN INJURY MARKERS (pg/mL)**						
**NfL**	-0.0014 (0.0035)	0.692	0.056	0.010 (0.017)	0.524	0.12
**GFAP**	-0.00041 (0.0015)	0.788	-0.030	-0.0011 (0.0024)	0.632	-0.084
**NEUROIMAGING**						
**Anterior cingulate cortex volume**	0.32 (0.10)**	0.00253	0.35	0.52 (0.20)*	0.0143	0.49

Supplementary Table 6: Linear regression models, variables included, and justification.

Model Name	Model Variables	Justification
Model 1: NeuroCOVID clinical	Global Deviation from Expected Score ~<br>Model foundation (Recruitment site, Days since COVID-19, Admission date, Interaction term: Days since COVID-19*Admission date)<br>+<br>Variables from Hypothesis 3 (Age, WHO COVID-19 severity, Diagnostic group, PHQ-9, Clinical Frailty Scale)<br>+<br>Variables from Hypothesis 4 (Level of education, Steroid treatment)	Clinical models contain predefined variables included in Hypothesis 3 (age, WHO COVID-19 disease severity, presence of an acute neurological or psychiatric complication, multimorbidity and mental health comorbidities, Rockwood clinical frailty scale) and Hypothesis 4 (level of education, and prior treatment with dexamethasone during acute illness) in the pre-registered statistical analysis plan. Acute serum inflammatory markers were excluded due to missingness >20%. Collinearity was assessed using correlation matrices. Fatigue, subjective cognitive impairment and mental health measures were found to be collinear. PHQ-9 score was considered most clinically significant based on existing literature and explained the most variance in GDfE and was therefore included in modelling (57). Included patients spanned 14 sites. Model adjusted for recruitment site, grouped as North (Liverpool, Newcastle, Salford Royal, Sheffield), South (Birmingham, Cambridge, Cardiff, Nottingham, Oxford, Southampton) and London (GSTT, KCH, SLaM, UCLH). Sites were grouped to avoid overfitting the effects of 14 individual centres, some of which only recruited a small number of participants. The interaction term was included because recovery trajectories may not be linear and may differ by epoch of illness.
Model 2: COVID clinical	Global Deviation from Expected Score ~<br>Model foundation (Recruitment site, Days since COVID-19, Admission date, Interaction term: Days since COVID-19*Admission date)<br>+<br>Variables from Hypothesis 3 (Age, WHO COVID-19 severity, PHQ-9, Clinical Frailty Scale)<br>+<br>Variables from Hypothesis 4 (Level of education, Steroid treatment)	As above.
Model 3: NeuroCOVID multifaceted model	Global Deviation from Expected Score ~<br>Model foundation (Recruitment site, Days since COVID-19, Admission date, Interaction term: Days since COVID-19*Admission date)<br>+<br>Clinical group (Diagnostic group)<br>+<br>Core clinical variables (Age, Multimorbidity, PHQ-9)<br>+<br>Brain injury biomarkers (GFAP, NfL)<br>+<br>Neuroimaging (Anterior cingulate cortex volume)	Multimorbidity and PHQ-9 were considered clinically significant based upon existing literature and the clinical models (73, 76).<br>NfL and GFAP were chosen to represent neuronal and glial injury respectively and have been shown to be raised in acute and post-acute COVID-19 (34, 35).<br>Longitudinal research using UK Biobank data reported volume loss in the anterior cingulate cortex and other limbic structures following mild SARS-CoV-2 infection. The ACC was chosen over other limbic structures based on existing literature suggesting structural changes after COVID-19 illness, and its implication in cognitive decline (14, 77).
Model 4: COVID multifaceted model	Global Deviation from Expected Score ~<br>Model foundation (Recruitment site, Days since COVID-19, Admission date, Interaction term: Days since COVID-19*Admission date)<br>+<br>Clinical group (WHO COVID-19 severity)<br>+<br>Core clinical variables (Age, Multimorbidity, PHQ-9)<br>+<br>Brain injury biomarkers (GFAP, NfL)<br>+<br>Neuroimaging (Anterior cingulate cortex volume)	As above. Clinical group is classified according to WHO COVID-19 disease severity rather than the neurological diagnostic subgroup.
Model 5: NeuroCOVID recovery	Recovery ~<br>Days since COVID-19<br>+<br>Clinical group (Diagnostic group)<br>+<br>Core clinical variables (Age, Multimorbidity, PHQ-9)<br>+<br>Brain injury biomarkers (GFAP, NfL)	Recovery is calculated as GDfE at follow-up 1 minus GDfE at post-acute assessment.<br>Sample size too small to include full model foundation.<br>Neuroimaging excluded due to missingness.
Model 6: COVID recovery	Recovery ~<br>Days since COVID-19<br>+<br>Clinical group (WHO COVID-19 severity)<br>+<br>Core clinical variables (Age, Multimorbidity, PHQ-9)<br>+<br>Brain injury biomarkers (GFAP, NfL)	As above

Supplementary Figure 1: Correlation matrix of numeric and ordinal variables included in modelling (two-tailed Spearman's, pairwise complete observations).

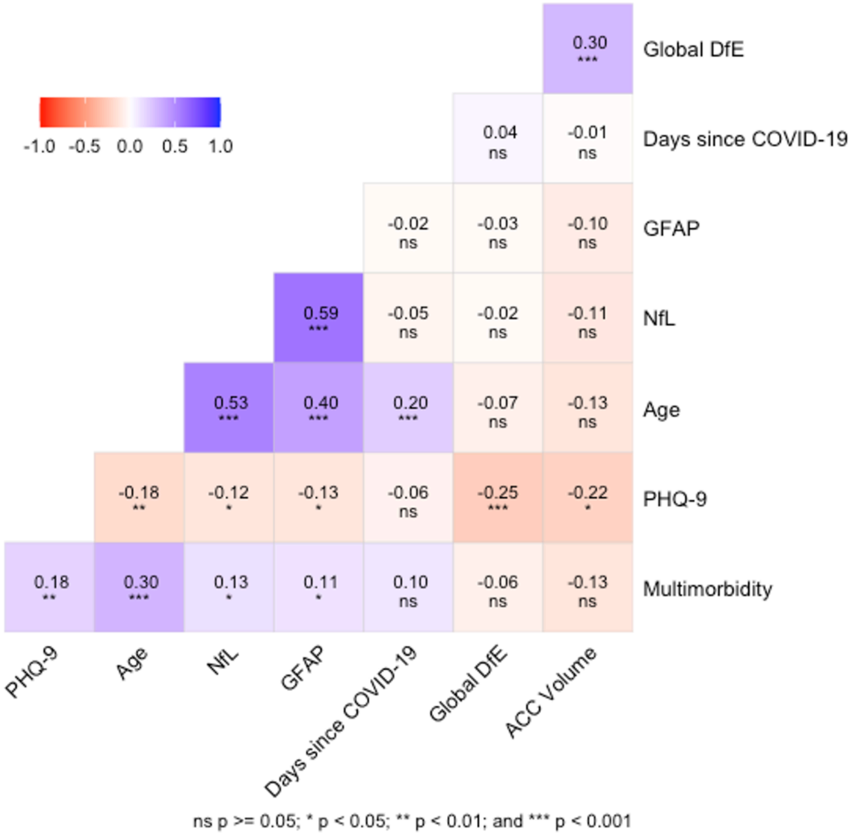

Supplementary Table 7: Detailing of analyses and application of correction for multiple comparisons based on false discovery rate (FDR) methodology. FDR was applied with a threshold of 5%.

**Analysis**	**FDR**	**Number of Comparisons**	**Justification**
Deviation from Expected Scores by Diagnostic Group	Yes	8	Two-sided Mann Whitney U across COVID, NeuroCOVID and 6 diagnostic subgroups
Pattern of deficits in clinical groups by median DfE accuracy and responsive time minus matched community controls across six cognitive tasks	Yes	12	Two-sided Mann Whitney U across 12 cognitron tasks within each diagnostic group
Recovery trajectories in NeuroCOVID and COVID patients following post-acute assessment	No	3	<4 comparisons: two-sided Mann Whitney U across baseline, follow-up 1 and follow-up 2 timepoints within COVID and NeuroCOVID group
Brain injury markers in pg/mL by diagnostic group.	No	3	<4 comparisons: two-sided Mann Whitney U across normative, COVID and NeuroCOVID groups within brain injury biomarkers (NfL, GFAP, Tau, UCH-L1)
Composite Image Derived Phenotypes against Deviation from Expected Scores	Yes	14	Shapiro-Wilk/Mann Whitney U, Linear Correlations and ANOVA across 14 image derived phenotype composites, within the COVID and NeuroCOVID group
Regression coefficients for Global DfE (GDfE) Score in NeuroCOVID group	No	31	Linear regression models of pre-specified candidate variables presented to allow comparison with clinical and multifaceted models (models 1-4). P values for these effect sizes presented but not used for interpretation of study findings.
Regression coefficients for Global DfE (GDfE) Score in COVID group	No	30	Linear regression models of pre-specified candidate variables presented to allow comparison with models 1-4. P values for these effect sizes presented but not used for interpretation of study findings.
Unsupervised cluster analysis across the full cohort	Yes	1369	Unsupervised cluster analysis between cognitive outcomes, pre-specified clinically important variables, brain injury biomarkers and neuroimaging image derived phenotypes across the full cohort of 351 patients: 1369 tests based on a 37x37 matrix of variables.

Supplementary Table 8. Exact p values for Figure 2a. Statistics compare each group to normative data. * p < 0.05, **p < 0.01, ***p < 0.001 two-sided Mann Whitney U, adjusted for multiple comparisons based on false discovery rate approach, n=8 comparisons. Median effect size = median DfE score of named diagnostic group subtract median DfE score of normative group.

	**Deviation from Expected (DfE) Score**	**Exact unadjusted p-value**	**Adjusted p-value**	**Significance marker**	**Median effect size**
**NeuroCOVID**	Global	5.22e-40	4.17e-39	***	-1.17
	Response Time	1.18e-24	9.46e-24	***	0.817
	Accuracy	1.42e-38	1.13e-37	***	-1.1
**COVID**	Global	6.06e-25	4.85e-24	***	-0.892
	Response Time	1.21e-15	9.65e-15	***	0.621
	Accuracy	5.14e-21	4.11e-20	***	-0.811
**Cerebrovascular**	Global	6.93e-11	5.55e-10	***	-1.26
	Response Time	2.87e-06	2.3e-05	***	0.988
	Accuracy	9.52e-12	7.62e-11	***	-1.29
**Inflammatory**	Global	2.89e-07	2.31e-06	***	-1.04
	Response Time	5.9e-05	0.000472	***	1.29
	Accuracy	1.43e-06	1.14e-05	***	-1.08
**Neuropsychiatric**	Global	1.97e-07	1.58e-06	***	-0.875
	Response Time	9.92e-05	0.000794	***	0.566
	Accuracy	8.47e-08	6.78e-07	***	-0.925
**Peripheral**	Global	1.86e-06	1.49e-05	***	-0.822
	Response Time	0.000161	0.00129	**	0.862
	Accuracy	7.91e-06	6.33e-05	***	-0.784
**Other**	Global	2e-08	1.6e-07	***	-0.982
	Response Time	9.43e-05	0.000755	***	0.664
	Accuracy	1.46e-08	1.17e-07	***	-1.12

Supplementary Table 9. Exact p values for Figure 2b. Statistics compare each group to normative data. * p < 0.05, **p < 0.01, ***p < 0.001 two-sided Mann Whitney U, adjusted for multiple comparisons based on false discovery rate approach, n=12 comparisons. Median effect size = median task DfE score of named diagnostic group subtract median task DfE score of normative group.

	**Task**	**Exact unadjusted p-value**	**Adjusted p-value**	**Significance marker**	**Median effect size**
**NeuroCOVID**	Recognition memory (immediate)	0.00016	0.00192	**	-0.294
	Spatial Span	6.41e-24	7.69e-23	***	-0.64
	2D manipulations	2.69e-24	3.22e-23	***	-0.698
	Verbal Analogies	2.59e-36	3.11e-35	***	-0.938
	Recognition memory (delayed)	0.00033	0.00396	**	-0.235
	Tower of London	1.54e-16	1.85e-15	***	-0.724
	Recognition memory (immediate) RT	6.47e-12	7.76e-11	***	0.461
	Spatial Span RT	1.93e-15	2.31e-14	***	0.62
	2D manipulations RT	4.93e-19	5.91e-18	***	0.603
	Verbal Analogies RT	9.77e-18	1.17e-16	***	0.66
	Recognition memory (delayed) RT	2.25e-07	2.7e-06	***	0.367
	Tower of London RT	0.000416	0.00499	**	0.289
**COVID**	Recognition memory (immediate)	0.0853	1		-0.169
	Spatial Span	1.17e-11	1.4e-10	***	-0.566
	2D manipulations	3.33e-13	3.99e-12	***	-0.498
	Verbal Analogies	1.82e-33	2.19e-32	***	-1
	Recognition memory (delayed)	0.548	1		0.0217
	Tower of London	3.18e-11	3.81e-10	***	-0.802
	Recognition memory (immediate) RT	6.07e-10	7.29e-09	***	0.474
	Spatial Span RT	7.29e-14	8.75e-13	***	0.698
	2D manipulations RT	9.91e-09	1.19e-07	***	0.477
	Verbal Analogies RT	2.76e-08	3.31e-07	***	0.393
	Recognition memory (delayed) RT	3.8e-05	0.000457	***	0.262
	Tower of London RT	0.417	1		0.0191
**Cerebrovascular**	Recognition memory (immediate)	0.0173	0.208		-0.684
	Spatial Span	1.4e-06	1.68e-05	***	-0.717
	2D manipulations	1.37e-08	1.64e-07	***	-1.05
	Verbal Analogies	2.39e-09	2.87e-08	***	-0.951
	Recognition memory (delayed)	0.00238	0.0285	*	-0.657
	Tower of London	9.26e-06	0.000111	***	-0.941
	Recognition memory (immediate) RT	0.0253	0.304		0.379
	Spatial Span RT	1.38e-05	0.000166	***	0.708
	2D manipulations RT	1.39e-06	1.66e-05	***	1.07
	Verbal Analogies RT	1.9e-05	0.000228	***	0.743
	Recognition memory (delayed) RT	0.159	1		0.285
	Tower of London RT	0.646	1		-0.188
**Encephalopathy**	Recognition memory (immediate)	0.0423	0.508		-0.558
	Spatial Span	1.82e-05	0.000218	***	-0.814
	2D manipulations	6.5e-08	7.8e-07	***	-0.776
	Verbal Analogies	3.7e-10	4.44e-09	***	-1.31
	Recognition memory (delayed)	0.382	1		0.0314
	Tower of London	0.000199	0.00239	**	-0.79
	Recognition memory (immediate) RT	1.08e-05	0.00013	***	0.983
	Spatial Span RT	0.000776	0.00931	**	0.635
	2D manipulations RT	9.9e-07	1.19e-05	***	0.942
	Verbal Analogies RT	2.37e-08	2.84e-07	***	1.08
	Recognition memory (delayed) RT	0.000245	0.00294	**	0.578
	Tower of London RT	0.000168	0.00201	**	0.693
**Inflammatory**	Recognition memory (immediate)	0.246	1		-0.257
	Spatial Span	2.16e-05	0.000259	***	-0.7
	2D manipulations	0.0893	1		-0.252
	Verbal Analogies	2.19e-08	2.63e-07	***	-1.5
	Recognition memory (delayed)	0.134	1		-0.255
	Tower of London	0.00875	0.105		-0.641
	Recognition memory (immediate) RT	0.000969	0.0116	*	0.489
	Spatial Span RT	0.000891	0.0107	*	0.808
	2D manipulations RT	0.186	1		0.174
	Verbal Analogies RT	0.0474	0.569		0.405
	Recognition memory (delayed) RT	0.0502	0.603		0.375
	Tower of London RT	0.117	1		0.469

**Neuropsychiatric**	Recognition memory (immediate)	0.234	1		-0.242
	Spatial Span	2.9e-05	0.000348	***	-0.476
	2D manipulations	3.26e-05	0.000392	***	-0.497
	Verbal Analogies	1.16e-07	1.4e-06	***	-1.04
	Recognition memory (delayed)	0.951	1		-0.185
	Tower of London	0.000834	0.01	*	-1
	Recognition memory (immediate) RT	0.00746	0.0895		0.408
	Spatial Span RT	0.000827	0.00993	**	0.553
	2D manipulations RT	0.000157	0.00189	**	0.505
	Verbal Analogies RT	0.000979	0.0117	*	0.449
	Recognition memory (delayed) RT	0.0274	0.329		0.196
	Tower of London RT	0.854	1		-0.012
**Peripheral**	Recognition memory (immediate)	0.176	1		-0.293
	Spatial Span	0.00232	0.0279	*	-0.445
	2D manipulations	0.000111	0.00134	**	-0.631
	Verbal Analogies	5.82e-05	0.000699	***	-0.439
	Recognition memory (delayed)	0.0541	0.65		-0.206
	Tower of London	0.00309	0.0371	*	-0.455
	Recognition memory (immediate) RT	0.0245	0.294		0.375
	Spatial Span RT	0.000259	0.0031	**	0.708
	2D manipulations RT	0.000246	0.00295	**	0.529
	Verbal Analogies RT	0.000172	0.00207	**	0.546
	Recognition memory (delayed) RT	0.137	1		0.178
	Tower of London RT	0.0124	0.148		0.332
**Other**	Recognition memory (immediate)	0.185	1		-0.106
	Spatial Span	8.89e-07	1.07e-05	***	-0.785
	2D manipulations	1.29e-05	0.000155	***	-0.776
	Verbal Analogies	1.79e-06	2.14e-05	***	-0.668
	Recognition memory (delayed)	0.103	1		-0.222
	Tower of London	0.000349	0.00419	**	-0.84
	Recognition memory (immediate) RT	0.00622	0.0747		0.466
	Spatial Span RT	0.0512	0.614		0.351
	2D manipulations RT	0.000412	0.00494	**	0.615
	Verbal Analogies RT	0.0124	0.148		0.484
	Recognition memory (delayed) RT	0.0131	0.158		0.496
	Tower of London RT	0.0525	0.63		0.583

Supplementary Information Only References

76. Russell CD, Lone NI, Baillie JK. Comorbidities, multimorbidity and COVID-19. Nat Med. 2023 Feb;29(2):334–43.

77. Sanabria-Diaz G, Etter MM, Melie-Garcia L, Lieb JM, Psychogios MN, Hutter G, et al. Brain cortical alterations in COVID-19 patients with neurological symptoms. Front Neurosci. 2022 Oct 20;16:992165.
