# Supplementary material for: Posthospitalization COVID-19 cognitive deficits at 1 year are global and associated with elevated brain injury markers and gray matter volume reduction
Source: Nat Med. 2024 Sep 23;31(1):245–57. doi: 10.1038/s41591-024-03309-8 (PMC11750706; doi:10.1038/s41591-024-03309-8)
Supplement: Supplementary file 2 — Reporting Summary [file 41591_2024_3309_MOESM2_ESM.pdf]

nature portfolio

Corresponding author(s): Prof Benedict Michael

Last updated by author(s): Aug 12, 2024

Reporting Summary

Nature Portfolio wishes to improve the reproducibility of the work that we publish. This form provides structure for consistency and transparency in reporting. For further information on Nature Portfolio policies, see our Editorial Policies and the Editorial Policy Checklist.

Statistics

For all statistical analyses, confirm that the following items are present in the figure legend, table legend, main text, or Methods section.

n/a	Confirmed	
☐	☒	The exact sample size (*n*) for each experimental group/condition, given as a discrete number and unit of measurement
☒	☐	A statement on whether measurements were taken from distinct samples or whether the same sample was measured repeatedly
☐	☒	The statistical test(s) used AND whether they are one- or two-sided<br>*Only common tests should be described solely by name; describe more complex techniques in the Methods section.*
☐	☒	A description of all covariates tested
☐	☒	A description of any assumptions or corrections, such as tests of normality and adjustment for multiple comparisons
☐	☒	A full description of the statistical parameters including central tendency (e.g. means) or other basic estimates (e.g. regression coefficient) AND variation (e.g. standard deviation) or associated estimates of uncertainty (e.g. confidence intervals)
☐	☒	For null hypothesis testing, the test statistic (e.g. *F*, *t*, *r*) with confidence intervals, effect sizes, degrees of freedom and *P* value noted<br>*Give P values as exact values whenever suitable.*
☒	☐	For Bayesian analysis, information on the choice of priors and Markov chain Monte Carlo settings
☒	☐	For hierarchical and complex designs, identification of the appropriate level for tests and full reporting of outcomes
☐	☒	Estimates of effect sizes (e.g. Cohen's *d*, Pearson's *r*), indicating how they were calculated

Our web collection on statistics for biologists contains articles on many of the points above.

Software and code

Policy information about availability of computer code

Data collection	Cognitive data was collected using Cognitron software. Brain injury markers were measured in serum using a Quanterix Simoa kit run on an SR-X Analyser (Quanterix, Billerica, MA, USA, Neurology 4-Plex A Advantage Kit, cat#102153). 3T MRI protocols were harmonized and the published standardised protocol was demonstrated to be consistent across sites (Duff et al). MRI data were processed with FSL (version 6.0) and Freesurfer (version 6.0), using the established UK Biobank pipeline modified for COVID-CNS (https://www.fmrib.ox.ac.uk/ukbiobank/fbp/). Clinical data was collected via Qualtrics from clinical notes.
Data analysis	Statistical analyses and data visualizations were performed in R (The R Foundation©, version 3.6.1 or later). Code will be made publicly available via https://github.com/tnggroup/covidcns

For manuscripts utilizing custom algorithms or software that are central to the research but not yet described in published literature, software must be made available to editors and reviewers. We strongly encourage code deposition in a community repository (e.g. GitHub). See the Nature Portfolio guidelines for submitting code & software for further information.

Data

Policy information about availability of data

All manuscripts must include a data availability statement. This statement should provide the following information, where applicable:

- Accession codes, unique identifiers, or web links for publicly available datasets
- A description of any restrictions on data availability
- For clinical datasets or third party data, please ensure that the statement adheres to our policy

Individual-level data and samples from the COVID-Clinical Neuroscience Study are available for collaborative research by application through the NIHR BioResource Data Access Committee https://bioresource.nihr.ac.uk/using-our-bioresource/apply-for-bioresource-data-access/. The Committee decide on academic applications, with escalation to the NIHR BioResource Steering Committee for contentious applications, and/or applications from industry. Participants in the NIHR BioResource have all consented to the sharing of de-identified data with bona fide researchers worldwide, for research in the public interest. There are limits to these consents both by expectation and legal - some datasets may not be shared beyond a safe setting in the UK. The Data Access Committee aim to process data-only requests as quickly as possible and meet fortnightly to consider applications. Once approved, timeframes for data availability vary from 2 weeks to 6 months depending on the nature of the data requested.

Human research participants

Policy information about studies involving human research participants and Sex and Gender in Research.

Reporting on sex and gender	Sex was reported based upon self-report. Raw Cognitron scores were transformed into Deviation from Expected (DfE) scores using established linear models trained on a large normative dataset designed to predict performance based upon age, sex, first language and level of education. There is no consent for sharing of individual level data to publish disaggregated, but sex has as such been evaluated in the group-wise aggregated data.
Population characteristics	As per Table 1 in full manuscript.<br>- Sex: NeuroCOVID group 118/190 (62%) male, COVID group 84/161 (52%) male.<br>- Age: NeuroCOVID group median age 54, COVID group median age 54.<br>- Level of Education: NeuroCOVID group 83/190 (44%) educated to college/university degree level, COVID group 83/161 (52%) educared to college/university degree level.<br>- WHO COVID-19 Severity: NeuroCOVID group 49/190 (31%) hospitalised severe, COVID group 40/161 (26%) hospitalised severe.<br>- COVID-19 vaccination status: NeuroCOVID group 31/190 (20%) vaccinated prior to study, COVID group 26/161 (18%) vaccinated prior to study.
Recruitment	Patients ≥16 years were recruited over 19 months (March 2021–Oct 2022) from 17 UK sites through the COVID-CNS, a case-control study within the National Institute of Health Research (NIHR) COVID-19 BioResource (REC reference 17/EE/0025; 22/EE/0230 (East of England—Cambridge Central Research Ethics Committee)). COVID-CNS included hospitalised patients with COVID-19 without a prior relevant neurological diagnosis, who have had a new acute neurological or psychiatric complication (NeuroCOVID) alongside COVID-19 controls without these diagnoses (COVID). NeuroCOVID patients were recruited if they met the prior published study-wide case definitions and inclusion/ exclusion criteria detailed in Supplementary Table 1. The NeuroCOVID group were identified by referral or admission to neurology, or by notification to the study team by the responsible clinician. Sites additionally screened relevant lists, for example, using clinical coding. The COVID group were recruited to match the NeuroCOVID group, matched on a group level by age, sex, ethnicity, pre-COVID clinical frailty status, COVID-19 severity, and epoch of admission during the pandemic. Admission dates were categorised into 6-month blocks as per input from the Infectious Diseases Experts at the National MRC Clinical Trials Unit and multidisciplinary Clinical Case Evaluation Panel, to reflect phases of the UK epidemic dominated by circulation of different SARS-CoV-2 variants, and changes in clinical practice. Some neurological or psychiatric complications required secondary care input without hospitalisation, partially related to pandemic pressures and risk assessments, and a proportion of the COVID group were therefore recruited who attended the emergency department but were not admitted. COVID-19 was defined by the WHO COVID-19 Case definition.
Ethics oversight	Participants were recruited into COVID-CNS and either the participant or their next of kin consented in accordance with the ethically-approved NIHR BioResource. (REC reference 17/EE/0025; 22/EE/0230 (East of England—Cambridge Central Research Ethics Committee)).

Note that full information on the approval of the study protocol must also be provided in the manuscript.

Field-specific reporting

Please select the one below that is the best fit for your research. If you are not sure, read the appropriate sections before making your selection.

☒ Life sciences ☐ Behavioural & social sciences ☐ Ecological, evolutionary & environmental sciences

For a reference copy of the document with all sections, see nature.com/documents/nr-reporting-summary-flat.pdf

Life sciences study design

All studies must disclose on these points even when the disclosure is negative.

Sample size	A sample size calculation in the pre-registered statistical analysis plan indicated that based on global G_SScore effect size and standard deviation published by Hampshire et al, we would require 129 patients from each group (NeuroCOVID and COVID) to have a 95% power at the 0.01 significant level (n=258) or 94 patients from each group to have a 95% power at the 0.05 significant level (n=188). We included n=351.
Data exclusions	Using a pre-established Cognitron processing pipeline, a technical correction was applied excluding those responding unfeasibly fast or slow based upon normative data. COVID-CNS clinical study wide inclusion/ exclusion criteria are included in Supplementary material. Patients with significant pre-existing neurological or psychiatric disorders managed in secondary care or pre-existing cognitive impairment were excluded. This formed part of the pre-existing COVID-CNS recruitment criteria.
Replication	Data analysis was conducted on clinical data, serum samples, and neuroimaging data collected as part of the COVID-CNS study. This is a relatively unique dataset and we do not have access to a second similar dataset to allow replication of study findings. We have referenced studies from which there are specific findings consistent with our analysis - including references 14, 30, 31, 32, 33, 38.
Randomization	N/A. This was an observational study.
Blinding	Serum samples were analysed with de-identified numeric identification numbers so that the researchers did not know what group they belonged to when assays were run.

Reporting for specific materials, systems and methods

We require information from authors about some types of materials, experimental systems and methods used in many studies. Here, indicate whether each material, system or method listed is relevant to your study. If you are not sure if a list item applies to your research, read the appropriate section before selecting a response.

Materials & experimental systems

n/a	Involved in the study	
☒	☐	Antibodies
☒	☐	Eukaryotic cell lines
☒	☐	Palaeontology and archaeology
☒	☐	Animals and other organisms
☒	☐	Clinical data
☒	☐	Dual use research of concern

Methods

n/a	Involved in the study	
☒	☐	ChIP-seq
☒	☐	Flow cytometry
☐	☒	MRI-based neuroimaging

Magnetic resonance imaging

Experimental design

Design type	n.a., T1 MPRAGE data only presented in this study. Additional MRI modalities (T2 FLAIR, resting state BOLD fMRI, Diffusion Weighted Imaging, Arterial Spin Labelling and Quantitative Susceptibility Mapping) were also acquired in the same scanning session but are not presented in this study so are not reported here.
Design specifications	T1mprage – GE scanner: 3Tesla MR 750<br>Scan plane: sagittal<br>Fov 25.6cm<br>Phase fov 1.0<br>Slice thick 1mm<br>Freq dir: S/I<br>Slabs: 1<br>Locs per slab: 208<br>No of TEs 1<br>TE min (1.9ms)<br>Flip angle 8deg<br>Prep time (YI): 800ms<br>Points in Frequency direction 256<br>Points in Phasedirection 256<br>Nex 1<br>BandWidth: 31.25<br>Aceleration: phase direction: 2.0, slice direction: 1.25<br>MPRAGE Repetition Time: (TR) = 2000<br>Acquisition time 4:42

Behavioral performance measures | No behavioural measures as part of scan sequence utilised.

Acquisition

Imaging type(s): Structural (only modality presented in this study) - T1 (MPRAGE). Additional MRI scans (T2 FLAIR, resting state BOLD fMRI, Diffusion Weighted Imaging, Arterial Spin Labelling and Quantitative Susceptibility Mapping) were also acquired in the same scanning session but are not presented in this study so are not reported here.

Field strength: 3T

Sequence & imaging parameters:
(for GE scanner)
T1 - TI/TR = 800/2000 ms, R = 2.
TE min (1.9ms)
Flip angle 8deg
Prep time (YI): 800ms
Points in Frequency direction 256
Points in Phasedirection 256
Nex 1
BandWidth: 31.25
Aceleration: phase direction: 2.0, slice direction: 1.25
MPRAGE Rpetition Time: (TR) = 2000
Acquisition time 4:42

Area of acquisition: Whole brain

Diffusion MRI ☐ Used ☒ Not used

Preprocessing

Preprocessing software: Each MRI modality was analysed using custom pipelines for image pre-processing and estimation of multiple MRI contrast metrics and imaging-derived phenotypes (IDPs), derived from the UKB analysis pipelines (www.fmrib.ox.ac.uk/ukbiobank/) and software tools from the FMRIB Software Library and FreeSurfer, with DICOM conversion carried out using DCM2NIIX.

Normalization: Processing of T1-weighted data included removal of the face, brain extraction, and registration to the MNI152 brain template (Jenkinson 2002, Andersson 2008).

Normalization template: MNI152

Noise and artifact removal: *Describe your procedure(s) for artifact and structured noise removal, specifying motion parameters, tissue signals and physiological signals (heart rate, respiration).*

Volume censoring: *Define your software and/or method and criteria for volume censoring, and state the extent of such censoring.*

Statistical modeling & inference

Model type and settings: Structural image derived phenotypes (IDPs) utilized in univariate and multivariate statistical tests. Global cognitive deviation from expected scores used as outcome in multivariate modeling.

Effect(s) tested: Structural data only presented in this study. No task-related design, so no effect tested as per this definition.

Specify type of analysis: ☐ Whole brain ☒ ROI-based ☐ Both

Anatomical location(s): Specific brain regions were selected based on extant literature a priori to analysis; the parahippocampal gyrus, entorhinal cortex, orbitofrontal cortex, anterior cingulate cortex, insula and superior temporal gyrus. MRI data were processed with FSL and Freesurfer, using the established UK Biobank pipeline, modified for COVID-CNS, in order to produce biologically relevant metrics of brain structure and function - IDPs. IDPs from T1 weighted MRI were obtained for global brain regions and for cortical regions as defined by Desikan-Killiany parcellation.

Statistic type for inference (See Eklund et al. 2016): Structural analyses only, use of IDPs as described.

Correction: FDR correction used in statistical reporting.

Models & analysis

n/a	Involved in the study
☒	☐ Functional and/or effective connectivity
☒	☐ Graph analysis
☒	☐ Multivariate modeling or predictive analysis
